# Pleiotropic Impact of Endosymbiont Load and Co-Occurrence in the Maize Weevil *Sitophilus zeamais*

**DOI:** 10.1371/journal.pone.0111396

**Published:** 2014-10-27

**Authors:** Gislaine A. Carvalho, Juliana L. Vieira, Marcelo M. Haro, Alberto S. Corrêa, Andrea Oliveira B. Ribon, Luiz Orlando de Oliveira, Raul Narciso C. Guedes

**Affiliations:** 1 Departamento de Bioquímica e Biologia Molecular, Universidade Federal de Viçosa, Viçosa, MG, Brazil; 2 Departamento de Entomologia, Universidade Federal de Viçosa, Viçosa, MG, Brazil; 3 Departamento de Entomologia e Acarologia, Escola Superior de Agricultura “Luiz de Queiroz”, Universidade de São Paulo, Piracicaba, São Paulo, Brazil; University of Innsbruck, Austria

## Abstract

Individual traits vary among and within populations, and the co-occurrence of different endosymbiont species within a host may take place under varying endosymbiont loads in each individual host. This makes the recognition of the potential impact of such endosymbiont associations in insect species difficult, particularly in insect pest species. The maize weevil, *Sitophilus zeamais* Motsch. (Coleoptera: Curculionidae), a key pest species of stored cereal grains, exhibits associations with two endosymbiotic bacteria: the obligatory endosymbiont SZPE (*“Sitophilus zeamais* Primary Endosymbiont”) and the facultative endosymbiont *Wolbachia*. The impact of the lack of SZPE in maize weevil physiology is the impairment of nutrient acquisition and energy metabolism, while *Wolbachia* is an important factor in reproductive incompatibility. However, the role of endosymbiont load and co-occurrence in insect behavior, grain consumption, body mass and subsequent reproductive factors has not yet been explored. Here we report on the impacts of co-occurrence and varying endosymbiont loads achieved via thermal treatment and antibiotic provision via ingested water in the maize weevil. SZPE exhibited strong effects on respiration rate, grain consumption and weevil body mass, with observed effects on weevil behavior, particularly flight activity, and potential consequences for the management of this pest species. *Wolbachia* directly favored weevil fertility and exhibited only mild indirect effects, usually enhancing the SZPE effect. SZPE suppression delayed weevil emergence, which reduced the insect population growth rate, and the thermal inactivation of both symbionts prevented insect reproduction. Such findings are likely important for strain divergences reported in the maize weevil and their control, aspects still deserving future attention.

## Introduction

Symbiosis is the result of intricate ecological relationships. Such intricacy may lead to shifts in the selection pressure over an organism, which may result in advantage or disadvantage to at least one of the interacting organisms of different species [1]–[3]. Intracellular bacteria are common endosymbionts of arthropods, either in obligatory or facultative associations, that live within the cells of their hosts [4], [5]. Not only nutrition-involved obligatory endosymbionts, such as *Buchnera* and *Wigglesworthia*, are of recognized importance in arthropods but also facultative endosymbionts, such as *Wolbachia, Hamiltonella,* and *Serratia,* among others [2], [3], [5]–[8]. Approximately 10% of insect species exhibit a primary (i.e., obligatory) endosymbiont, while an estimated 40% of insect species host some *Wolbachia* strain [9], [10].

The specialized and unbalanced diets of several arthropod species is an indication of the potential importance of their endosymbionts, which frequently play a fundamental role in complementing nutrition in their host, allowing host survival in novel environments and under alternate food source [2], [3], [6]–[8], [9]. Although such a role is likely a pivotal innovation in arthropod evolution, the specific roles of the majority of their endosymbionts remains unknown [2], [3], [9]. The suppression or inactivation of endosymbionts shed some light on this matter, as exemplified by the *Wolbachia*-mediated fitness increase and parasitism protection of whiteflies [11], and high temperature tolerance and parasitoid resistance provided by *Serratia* and *Hamiltonella* [12]–[15].

Understanding the role of endosymbionts in the behavioral, ecological and evolutionary processes of arthropods is no easy task. This is so not only because of individual trait variation within an arthropod population [16] but also because an arthropod may host varying loads of more than one endosymbiont, confounding and/or masking their impact and importance in the host individual. Weevils in the genus *Sitophilus*, which encompasses three grain weevil species of key importance for stored grain protection (*Sitophilus granarius, S. oryzae,* and *S. zeamais*), host both primary (obligatory) and secondary (facultative) endosymbionts, making then suitable models to study the roles of co-existing symbionts and their eventual relevance for pest control [17]–[23].

Grain weevils exploit a restrictive food source, cereal grains, and must complete their development within the grain kernel. The association between grain weevils and their primary endosymbiont SPE (*Sitophilus* Primary Endosymbiont) is hypothesized to be an important requirement allowing survival under such conditions [19], [23], [24]. However, physiological differences do exist among weevil strains, allowing strain variation in how well they are able to cope with cereal amylase inhibitors and insecticide exposure [25], [26]. SPE was initially detected in the rice weevil (*S. oryzae*), where it is referred to as SOPE (*Sitophilus oryzae* Primary Endosymbiont; = *Candidatus* Sodalis pierantonius str. SOPE), and subsequently in the granary and maize weevils (*S. granarius* and *S. zeamais*), where it is referred to as SGPE (*Sitophilus granarius* Primary Endosymbiont; = *Candidatus* Sodalis pierantonius) and SZPE (*Sitophilus zeamais* Primary Endosymbiont; = *Candidatus* Sodalis pierantonius str. SZPE) [11]–[12], [18]–[19], [27], respectively.

SPE seems to provide vitamins to its weevil hosts, assisting in their amino acid metabolism, in addition to interacting with mitochondrial oxidative phosphorylation, thus enhancing respiration and mitochondrial enzyme activity in the host insect [17], [28]–[30]. Such effects of SPE may affect development, immune response and flight activity in their weevil hosts [17], [31]. Curiously, however, the focus of previous SPE studies has remained on the genetics and molecular biology of these endosymbionts [18], [32]–[35], and not on their behavioral or physiological consequences in the weevil hosts. However, the co-occurrence of SPE and *Wolbachia* in cereal weevils [18]–[20], raises questions regarding their interaction and potential impact on this host species. Here we recognized the presence of both SZPE and *Wolbachia* in the maize weevil, subjected the colonized weevil hosts to different treatments for endosymbiont inactivation/suppression, assessed the impacts of endosymbiont loads of either one or both symbionts, and analyzed how they affect host reproductive fitness following a structured hierarchical approach. Past studies focused on the simultaneous presence/absence of such endosymbionts [17], [28]–[30], while here presence was quantified and associated with behavioral and physiological traits potentially affecting the insect reproductive output.

## Materials and Methods

### Ethic Statement

This study did not involve any endangered or protected species. The insect species studied is a cereal pest species from a colony maintained in laboratory, where the experiments were performed, and no specific permission was required.

### Insects

The insects were obtained from an insecticide-susceptible laboratory colony of the maize weevil (*S. zeamais*) that has been maintained in whole maize kernels free of insecticide residues since the mid-1980s [16], [36], [37]. The insects are maintained under controlled conditions of 27±2°C temperature, 70±10% relative humidity, and a 12 h photoperiod, the same conditions employed in our bioassays.

**Figure 3:**
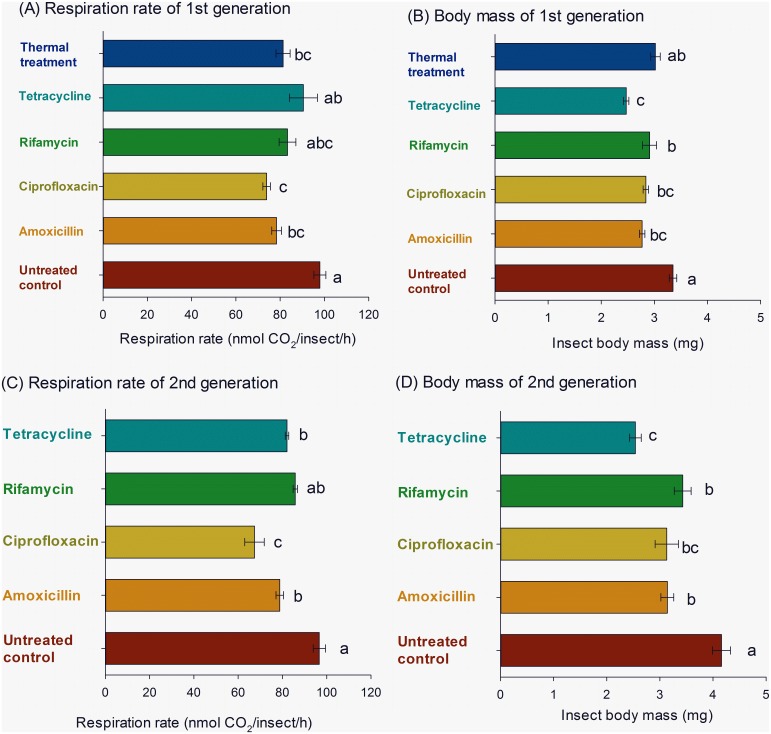
Respiration rate (A, C) and body mass (B, D) (± SE) of F_1_ (A, B) and F_2_ progenies (C, D) of maize weevils (*Sitophilus zeamais*) exposed to different endosymbiont-reducing treatments. Means followed by the same letter in a histogram are not significantly different by Tukey’s HSD test (*P*<0.05).

### Endosymbiont Quantification and Inactivation/Suppression

#### PCR amplification for endosymbiont load quantification

The endosymbiont load in individual adult weevils was quantified using quantitative polymerase chain reaction (qPCR) after individual DNA extraction. The total genomic DNA of adult maize weevils (> one-week old) was extracted following Clark [38]. PCR amplification was performed in a total volume of 12 µL, and consisted of 1 µL DNA, 6.0 µL SYBR Green Master Mix (2x; Applied Biosystems, Foster City, CA, USA), and 200 nM of each primer (forward [F] and reverse [R] primers for the 16S rRNA gene of SZPE and for the 16S rRNA gene of *Wolbachia*). The following sets of primers were used: (1) 5′-AGACTCTAGCCTGCCAGTTT-3′ (F primer) and 5′-AGCTGTAATACAGAAAGTAAA-3′ (R primer) for the 16S rRNA of SZPE, generating a 145 bp DNA fragment; and (2) 5′-CGGGGGAAAAATTTATTGCT-3′ (F primer) and 5′-TAGGAGTCTGGACCGTATCT-3′ (R primer) for the 16S rRNA of *Wolbachia*, generating a 198 bp DNA fragment. The design of the oligonucleotide pairs was performed using Primer3 Plus software [39], following the requirements of real time PCR. No-template controls, containing nuclease-free water, were included in each run.

The PCR was performed on an ABI Prism 7500 Sequence Detection System (Applied Biosystems, Foster City, CA, USA). The PCR cycles used the following conditions: 2 min at 50°C followed by 10 min denaturation at 95°C, 40 cycles of 45 s denaturation at 95°C, and annealing and extension at 60°C for 30 s. After the 40 cycles of amplification, all of the samples were subjected to gradual denaturation to elaborate the dissociation curve. The samples were heated at 1°C increments every 30 s from 60 to 94°C. The melting curves (65°C to 97°C) were obtained at the end of each reaction to ascertain the specificity of the PCR product. The standard curve was plotted using the following eight dilutions of the corresponding plasmids of each gene fragment: 2.92×10^1^, 2.92×10^2^, 2.92×10^3^, 2.92×10^4^, 5.03×10^4^, 1.08×10^6^, 2.08×10^6^, and 3.08×10^6^ copies/µL. The number of fragment copies of each gene was estimated using the standard curve, and the amount of total DNA (host+endosymbionts) in each sample was used to standardize the results of the number of copies of the 16S rRNA gene fragments [40]. The one-point calibration method (OPC) was used to correct the obtained values, minimizing the differences between plasmid and total DNA [41]. The results were presented as number of copies per ng of DNA. Three independent biological samples were analyzed in triplicate, and their endosymbiont load was quantified in independent amplifications. The same methods for quantifying endosymbiont load were performed on the adult progeny of adult weevils subjected to each of the different endosymbiont suppression treatments, in addition to adult weevils without such suppression (control).

#### Endosymbiont reduction

Two approaches were used for endosymbiont load reduction: inactivation via thermal treatment and suppression via antibiotic ingestion. The thermal treatment was based on the exposure of adult weevils to high temperature and humidity [19]. For this purpose, maize weevil adults (over one week old) were transferred to transparent plastic containers (250 mL) half-filled with whole maize grains and maintained for 21 days in an environmental chamber under controlled conditions of 37±2°C and 90±5% relative humidity.

Endosymbiont suppression in adult weevils was performed by providing antibiotics through ingested water to insects subjected to 24 h of hydric stress. Hydric stress was achieved by individually containing one-week old adults in perforated Eppendorf tubes placed within glass desiccators (3,000 cm^3^) at 1% relative humidity (27±2°C and 12 h photoperiod) for 24 h, after which they avidly ingest water from water droplets [42]. The weevils were subsequently transferred individually to Petri dishes (9 cm diameter) containing a 5 µL droplet of water-diluted antibiotic (either amoxicillin, ciprofloxacin, rifamycin, or tetracycline, at 25 mg/mL). The antibiotics were obtained from Medley (Campinas, SP, Brazil), Genfar (Bogotá, Colômbia), Legrand (Campinas, SP, Brazil), and Bristol-Myers Squibb (São Paulo, SP, Brasil), respectively, at their available commercial formulations (Amoxicilina 250 mg, Ciprofloxacino 500 mg, Rifamicina 10 mg/ml, Tetrex 500 mg). The antibiotic concentration used was established after preliminary concentration-response bioassays using the following range of concentrations: 0, 1, 5, 10, 25, 50 and 100 mg/mL.

The insects were maintained for 40 min in the Petri dishes with the desired water-diluted antibiotic and subsequently transferred to maize contained in Petri dishes for 24 h; this procedure was repeated six times for each individual insect. The progeny of the treated insects was also subjected to the same antibiotic treatment. Therefore the antibiotic-treated insects were from the parental (P) generation when the F_1_ progeny was assessed, and from the P and F_1_ generations when the F_2_ progeny was assessed. Only the progenies of the insects treated for one or two generations were used in the endosymbiont quantification and subsequent bioassays in order to eliminate the eventual deleterious effects of the antibiotics themselves on insect performance. This was not possible for the thermal treatment because the treated (parental) weevil generation was unable to reproduce and the treated insects themselves were therefore used in the subsequent bioassays.

### Behavioral Bioassays

Four batches of 10 adult weevils (> one week old) from each endosymbiont inactivation/suppression treatment were subjected to six behavioral bioassays assessing overall insect activity, walking activity, flight activity (take-off and free-fall flight), body righting, and death-feigning. The methods for determining overall insect activity were adapted from Tomé et al. [43], while those for the remaining bioassays were adapted from Morales et al. [16]. All methods are briefly described below.

#### Overall group insect activity

The batches of 10 adult weevils were transferred to a Petri dish arena (9 cm diameter) with its bottom covered with filter paper (Whatman no. 1), allowing for better traction when walking and contrast for activity determination, and its inner walls were coated with Teflon PTFE (DuPont, Wilmington, DE, USA) to prevent the insects from escaping. The overall insect activity in each Petri dish arena, including walking behavior, insect interactions, and body part movements, were recorded for 10 min and digitally transferred to a computer using a video tracking system equipped with a digital CCD camera (ViewPoint LifeSciences, Montreal, QC, Canada). The overall insect activity was recorded as changes in pixels/s×10^−2^.

#### Walking activity

Walking activity was recorded for individual insects for 10 min following their release into Petri dish arenas prepared as previously described. A single insect was released in the center of the arena and its movement was recorded using the same tracking system used in the assessment of overall group activity. The following characteristics were evaluated: distance walked (cm), walking velocity (cm/s), and resting time (s).

#### Take-off flight

A hand-made wooden square box (18 cm wide, 18 cm deep, 30 cm high) covered with a 2 mm steel frame was used. Groups of 10 adult insects were placed at the central bottom of the box within an open Petri dish (5 cm diameter) with its bottom covered with a piece of filter paper (Whatman no. 1) and its inner walls coated with Teflon PTFE. The length of time for the insects to take off for flight, the number of insects entering flight, and heights reached in flight during 10 min trials were recorded.

#### Free-fall flight

A hand-made wooden square box (44 cm wide, 44 cm deep, 88 cm high) with its top covered with organza tissue with a 5 cm-diameter hole in the top center was used. A chalk-covered funnel was inserted in the central hole at the top of the wooden box. The box was placed on a marked sheet of paper with concentric circles spaced 3 cm apart for one another. Each adult weevil was placed in the upper central funnel of the wooden box, and its landing site was recorded by determining its distance from the center. Each insect was released three times and the average distance of flight was determined.

#### Body righting

Each adult weevil was placed on its dorsum and the time taken to recover its regular ventral posture was recorded. The procedure was replicated three times, and the average determination was recorded.

#### Death-feigning

Death-feigning induction was performed by dorsally prodding the adult weevil with a fine-haired brush and recording the time taken for the insect to start moving after reaching its typical death-feigning (or thanatosis) posture. The procedure was replicated three times and the average determination was used as the duration of the death-feigning behavior.

### Respiration Rate and Body Mass

The respirometry bioassays were carried out in a TR3C respirometer equipped with a CO_2_ analyzer (Sable Systems International, Las Vegas, NV, USA), as detailed elsewhere [44], [45]. Briefly, four replicates of 10 adult weevils from each endosymbiont-suppression treatment were gathered, and the insect body mass was determined with an analytical balance (Shimadzu AUW220D, Kyoto, Japan). The groups of 10 insects were subsequently contained in 25 mL glass respirometric chambers connected to a completely closed system. The CO_2_ produced by the insects (µL CO_2_/h) was determined by injecting CO_2_-free air into the chambers and directing the insect-produced CO_2_ to an infrared reader connected to the system. The CO_2_ production in a control chamber without insects was also determined.

### Developmental Rate and Grain Consumption

The experiment was performed using 1.0 L glass jars containing 300 g of whole maize. Ten adult couples of the maize weevil were released in each jar and removed 30 days later following methods by Trematerra et al. [46] and Fragoso et al. [47]. The daily and cumulative progeny emergence was assessed every other day, with four replicates (i.e., jars with ten couples and 300 g maize) for each endosymbiont-suppression treatment. The mass of grain consumption in each jar (i.e., replicate) was also determined at the end of the experiments when no more progeny emerged, 70 days after the experiment began.

### Statistical Analyses

Endosymbiont load, respiration rate, adult weevil body mass, and grain consumption were subjected to analyses of variance and Tukey’s HSD test when appropriate (PROC GLM; SAS v. 9) [48]. A canonical variate analysis (CVA) of the behavioral traits of weevils subjected to the different endosymbiont-suppression treatments was performed to recognize their eventual differences and the main contributing traits for observed differences (PROC CANDISC with Distance statement; SAS v. 9) [48]. Such behavioral results were subsequently subjected to complementary analysis of variance for the individual traits assessed and Tukey’s HDS test, if appropriate (PROC GLM; SAS v. 9) [48]. The normality and homoscedasticity assumptions were checked (PROC UNIVARIATE; SAS v. 9) [48], and log (x+1) transformation was necessary to stabilize the variance for the height of the flight take-off bioassay.

The daily and cumulative emergence results of weevils whose parental generation was subjected to the different endosymbiont-suppression treatments were subjected to non-linear regression analysis using the curve-fitting procedure of TableCurve 2D (Systat, San Jose, CA, USA). The significant regression models (*P*<0.05) were tested from the simplest (linear and quadratic) to more complex (peak and asymptotic) models basing the model selection on parsimony, high F-values (and mean squares), and a steep increase in R^2^ with model complexity. Residual distribution was also checked for each analysis to validate parametric assumptions.

Path analysis was used to test the hypothesized relationships between endosymbiont load in host weevils of the F_1_ progeny of the antibiotic-treated insects and potential direct and indirect consequences (including behavioral traits, respiration rate, body mass, grain consumption) potentially contributing to their progeny production. Only the data from endosymbiont suppression with antibiotic treatments was used in this analysis because the thermal treatment prevented assessment of the progeny of treated insects. This analysis was performed used the procedures PROC REG and PROC CALIS from SAS v. 9 [48], following guidelines provided by Mitchell [49].

## Results

### Endosymbiont Load and Reduction

**Figure 1:**
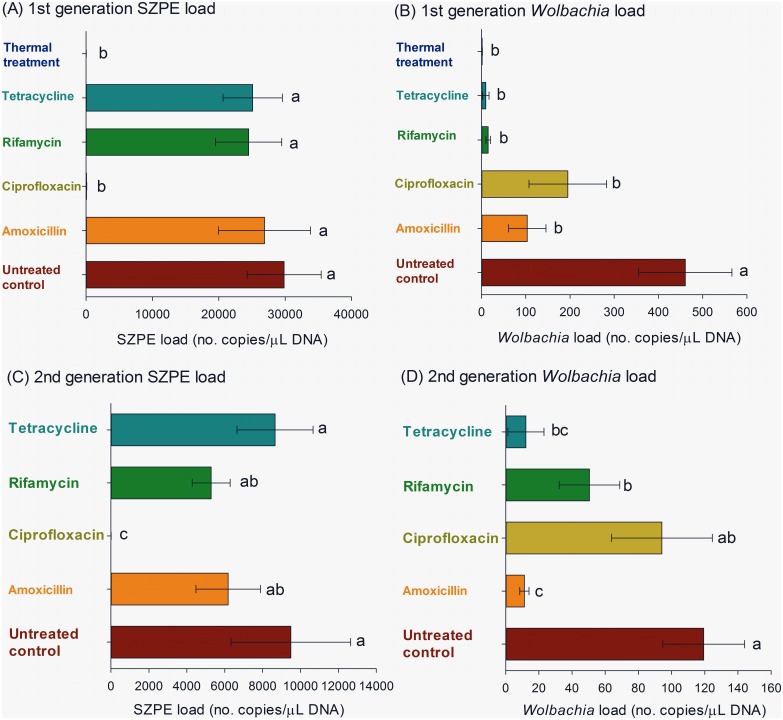
Load (± SE) of the endosymbionts SZPE (A, C) and *Wolbachia* (B, D) in F_1_ (A, B) and F_2_ progenies (C, D) of maize weevils (*Sitophilus zeamais*) exposed to different endosymbiont-reducing treatments. Means followed by the same letter in a histogram are not significantly different by Tukey’s HSD test (*P*<0.05).

The adult weevils subjected to the thermal treatment for endosymbiont inactivation and the F_1_ and F_2_ progenies of weevils subjected to antibiotic treatment for endosymbiont suppression were used to detect and quantify symbiont load based on the quantification of copy numbers of 16S rRNA gene fragments from SZPE and *Wolbachia*. The thermal treatment and the antibiotic ciprofloxacin were particularly effective in reducing the load of SZPE, while all antibiotics and the thermal treatment led to similar and significant reduction of *Wolbachia* loads in the F_1_ weevil progeny (Figs. 1A and 1B).

The adult weevils that were subjected to the thermal treatment, and consequently full inactivation of both SZPE and *Wolbachia*, were unable to reproduce. Therefore, the endosymbiont load in the subsequent progeny was not determined for this endosymbiont-inactivation treatment. In the treatments with antibiotics, ciprofloxacin obtained complete suppression of SZPE, tetracycline did not obtain significant suppression, and amoxicillin and rifamycin exhibited intermediate results (Fig. 1C). Amoxicillin obtained significantly higher levels of *Wolbachia* suppression (i.e., lower load of *Wolbachia*) followed by tetracycline, rifamycin, and ciprofloxacin, which exhibited similar levels of *Wolbachia* suppression (Fig. 1D).

### Behavioral Consequences of Endosymbiont Load

A multivariate analysis of variance performed with the CVA protocol from SAS indicated a significant overall effect of the endosymbiont-reducing treatments on the behavior of the F_1_ progeny of antibiotic-exposed weevils and thermally treated weevils (Wilks’ lambda = 0.0002, F = 8.29, d.f._num/den_ = 40/50, *P*<0.001). Subsequent (univariate) analyses of variance performed for each behavioral trait assessed indicated that symbiont reduction affected all behavioral traits except resting time (Table 1). The behavioral alterations caused by ciprofloxacin and thermal endosymbiont reduction are particularly noteworthy (Table 1).

**Table 1:** Behavioral traits (± SE) of maize weevils (*Sitophilus zeamais*) exposed to different endosymbiont-reducing treatments.

Treatment	Overallgroupactivity(Δ pixels/s×10^−2^)	Walking activity		Flight activity			Duration ofdeath-feigning (s)	Lengthof time tobodyrighting (s)
		Walkingvelocity (cm/s)	Restingtime (s)	HorizontalDislocationuponfall (cm)	No. takingoff forflight	Flightheightreached ontake-off (cm)		
Untreated control	45.85±2.86 a	0.41±0.01 a	227.11±9.71	40.41±6.17 a	3.25±0.25 a	13.29±1.7 a	5.61±0.89 bc	3.38±0.35 b
Amoxicillin	27.81±2.13 b	0.37±0.02 bc	246.92±11.84	7.00±0.24 b	0.51±0.29 bc	0.75±0.05 bc	6.41±0.56 bc	4.41±0.41 b
Ciprofloxacin	17.42±0.73 cd	0.34±0.01 c	265.06±9.55	5.35±0.24 b	0.00±0.00 c	0.00±0.00 c	15.93±1.17 a	7.56±0.52 a
Rifamycin	25.37±1.12 bc	0.35±0.02 bc	268.63±11.94	9.53±1.01 b	1.25±0.48 b	4.38±1.72 b	5.42±0.40 bc	3.58±0.25 b
Tetracycline	22.48±3.75 bc	0.33±0.01 c	269.48±7.66	7.17±0.39 b	0.25±0.25 bc	0.25±0.25 c	3.33±0.19 c	3.41±0.24 b
Thermal treatment	11.54±1.51d	0.31±0.01 c	280.26±11.17	5.75±0.26 b	0.00±0.00 c	0.00±0.00 c	7.86±0.95 b	6.65±0.46 a
F_5,18_	26.77	4.65	2.61	160.34	21.51	18.80	19.44	25.27
*P*	<0.001*	0.007*	0.06	<0.001*	<0.001*	<0.001*	<0.001*	<0.001*

Means followed by the same letter in a column are not significantly different by Tukey’s HSD test (*P*<0.05). Asterisks indicate significant differences among treatments by Fisher’s F test from the (univariate) analyses of variance for each behavioral trait.

**Figure 2:**
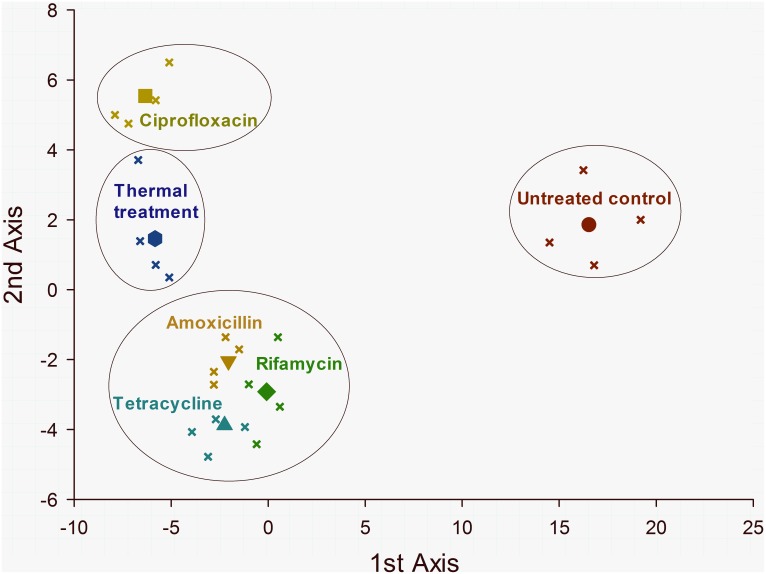
Ordination (CVA) diagram showing the divergence in behavioral traits of maize weevils (*Sitophilus zeamais*) exposed to different endosymbiont-reducing treatments (see Table 2). Both canonical axes are significant and account for 97.45% of the total variance explained. The solid symbols are centroids of treatments representing the class mean canonical variates and the smaller symbols of the same color represent the individual replicates. The large circles indicate clusters of treatments that are not significantly different by the approximated F-test (*P<0.05)*, based on the Mahalanobis (D^2^) distance between class means.

The multidimensional behavioral construct obtained with the CVA analysis representing the behavioral consequences of endosymbiont reduction in the maize weevil provided significant overall results. The CVA ordination generated five axes, of which the two first (1^st^ and 2^nd^) were significant (*P*<0.001), explaining 97.45% of the observed variance (Table 2). The number of insects taking off for flight and the horizontal dislocation upon free-fall flight followed by the flight height exhibited the greatest canonical loads for the 1^st^ axis accounting for most of the observed divergence among endosymbiont-reducing treatments, followed by the duration of death-feigning and the length of time to upturn, which accounted for most of the divergence on the 2^nd^ axis (Table 2). The CVA diagram derived from the CVA representing the maximum divergence in behavior among endosymbiont-reducing treatments emphasizes the differences in the thermal and ciprofloxacin treatments, with the other antibiotics exhibiting similar intermediate differences relative to the untreated weevils retaining their regular endosymbiont load (Fig. 2).

**Table 2:** Canonical loadings (between canonical structure) of the canonical axes for the behavioral traits of maize weevils (*Sitophilus zeamais*) exposed to different endosymbiont-reducing treatments.

Behavioral traits		Canonical axes				
		1^st^	2^nd^	3^rd^	4^th^	5^th^
Overall group activity (Δ pixels/s×10^−2^)		**0.90**	−0.09	0.33	−0.17	0.03
Walking activity	Walking velocity (cm/s)	0.67	0.02	0.39	−0.14	0.29
	Resting time (s)	−0.55	**0.67**	−0.31	−0.24	−0.28
Flight activity	Horizontal dislocation upon fall (cm)	**0.91**	0.17	−0.10	−0.02	0.01
	No. taking off for flight	**0.92**	0.01	0.08	0.23	−0.01
	Flight height reached on take-off (cm)	**0.87**	−0.04	0.15	0.39	−0.05
Duration of death-feigning (s)		−0.37	**0.82**	0.34	0.02	−0.20
Length of time to body righting (s)		−0.58	**0.75**	−0.10	0.04	0.25
F*_appr._*		8.29	3.83	1.69	1.14	1.02
*P*		<0.001*	<0.001*	0.09	0.23	0.43
Eigenvalue		79.47	14.22	1.40	0.77	0.27

Bold type indicates the main contributors of each axis and asterisks indicate the significant axes.

### Respiration Rate, Body Mass, and Grain Consumption

**Figure 4:**
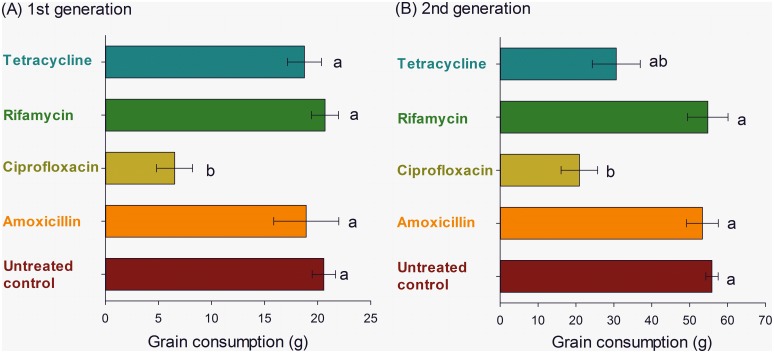
Grain consumption (± SE) of F_1_ (A) and F_2_ progenies (B) of maize weevils (*Sitophilus zeamais*) exposed to different endosymbiont-reducing treatments. Means followed by the same letter in a histogram are not significantly different by Tukey’s HSD test (*P*<0.05).

Weevil respiration rate varied significantly among the endosymbiont-reducing treatments (F_5,18_ = 5.72, *P* = 0.002), with all treatments except rifamycin and tetracycline leading to significant reduction relative to the control (Fig. 3AC). Body mass followed a trend similar to respiration rate (F_5,18_ = 12.81, *P*<0.001), but the F_1_ progeny of antibiotic-treated insects exhibited lower body mass (Fig. 3BD). Grain consumption also differed significantly among F_1_ progeny weevils of endosymbiont-reduced parental insects (F_4,15_ = 10.15, *P*<0.001), with the F_1_ progeny of ciprofloxacin-treated parents exhibiting the lowest levels of grain consumption (Fig. 4A). The results obtained with the F_2_ progeny of the endosymbiont-reduced insects were also significant (*P*<0.003) and largely congruent with the results from the F_1_ progeny (Fig. 4B).

### Daily and Cumulative Emergence

**Figure 5:**
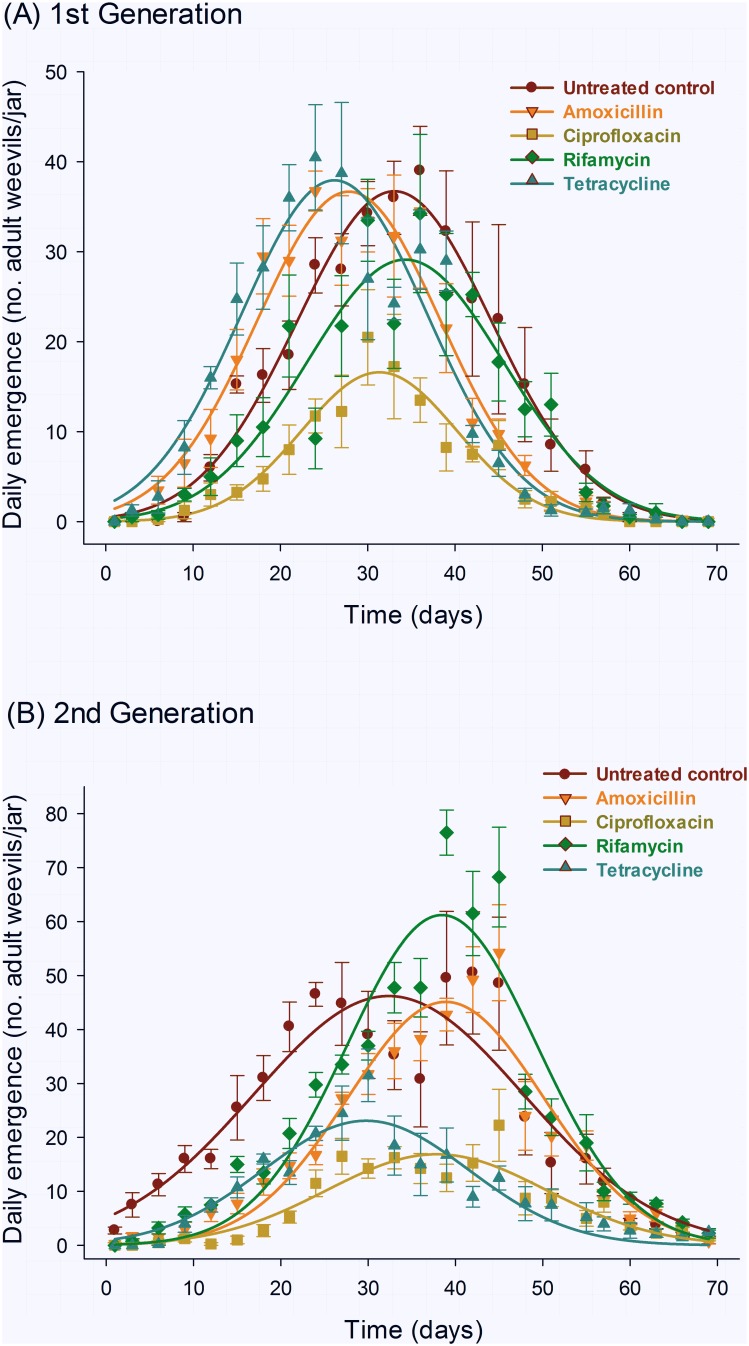
Daily emergence of F_1_ (A) and F_2_ progenies (B) of maize weevils (*Sitophilus zeamais*) exposed to different endosymbiont-reducing treatments. The symbols and vertical bars represent the means and standard errors of four replicates and the equation parameters are exhibited in Table 3.

The profile of daily adult emergence of the F_1_ and F_2_ progenies of parental weevils subjected to endosymbiont suppression markedly differed among treatments, and followed the three-parameter Gaussian model used to describe the trend and selected as previously described (Table 3). Amoxicillin and tetracycline, although advancing the emergence peak of F_1_ progenies, compromised adult emergence in the F_2_ progeny but not in the F_1_ progeny (Fig. 5). In contrast, rifamycin slightly delayed the peak of adult emergence relative to the control, reducing it for the F_2_ progeny, but increasing the peak of emergence for the F_2_ progeny when compared with the control (Fig. 5). Additionally, the ciprofloxacin endosymbiont-reduced progeny of treated parental weevils exhibited longer delays the for F_2_ progeny, and reduced peaks of adult emergence for both F_1_ and F_2_ progenies (Fig. 5).

**Table 3:** Summary of the non-linear regression analyses of the daily emergence curves (Fig. 5) of the F_1_ and F_2_ progenies of adult maize weevils (*Sitophilus zeamais*) exposed to different endosymbiont-suppression treatments via water-ingested antibiotics.

Generation	Model	Treatment	Parameter estimates (± SE)			df_error_	F	*P*	R^2^
			*a*	*b*	*c*				
		Untreated control	36.73±1.76	33.05±0.62	11.13±0.62	93	154.51	<0.001	0.76
	Gaussian (3-parameter)	Amoxicillin	36.68±1.56	27.84±0.52	10.55±0.52	93	203.18	<0.001	0.81
1^st^	y = *a* exp(−0.5((x–*b*)/*c*)^2^)	Ciprofloxacin	16.60±1.11	31.32±0.69	8.93±0.69	93	89.03	<0.001	0.65
		Rifamycin	29.13±1.75	34.30±0.79	11.30±0.79	93	94.97	<0.001	0.67
		Tetracycline	37.95±1.65	26.17±0.54	10.67±0.54	93	196.25	<0.001	0.81
		Untreated control	46.20±2.57	32.35±0.99	15.43±1.02	93	72.25	<0.001	0.61
	Gaussian (3-parameter)	Amoxicillin	45.12±1.80	38.89±0.52	11.22±0.51	93	201.32	<0.001	0.80
2^nd^	y = *a* exp(−0.5((x–*b*)/*c*)^2^)	Ciprofloxacin	16.88±1.05	37.85±0.91	12.58±0.91	93	77.11	<0.001	0.62
		Rifamycin	61.21±2.32	38.47±0.48	10.87±0.47	93	223.09	<0.001	0.83
		Tetracycline	23.09±1.35	29.76±0.80	11.76±0.80	93	84.06	<0.001	0.64

All parameter estimates were significant at *P*<0.01 by Student’s *t*-test.

**Figure 6:**
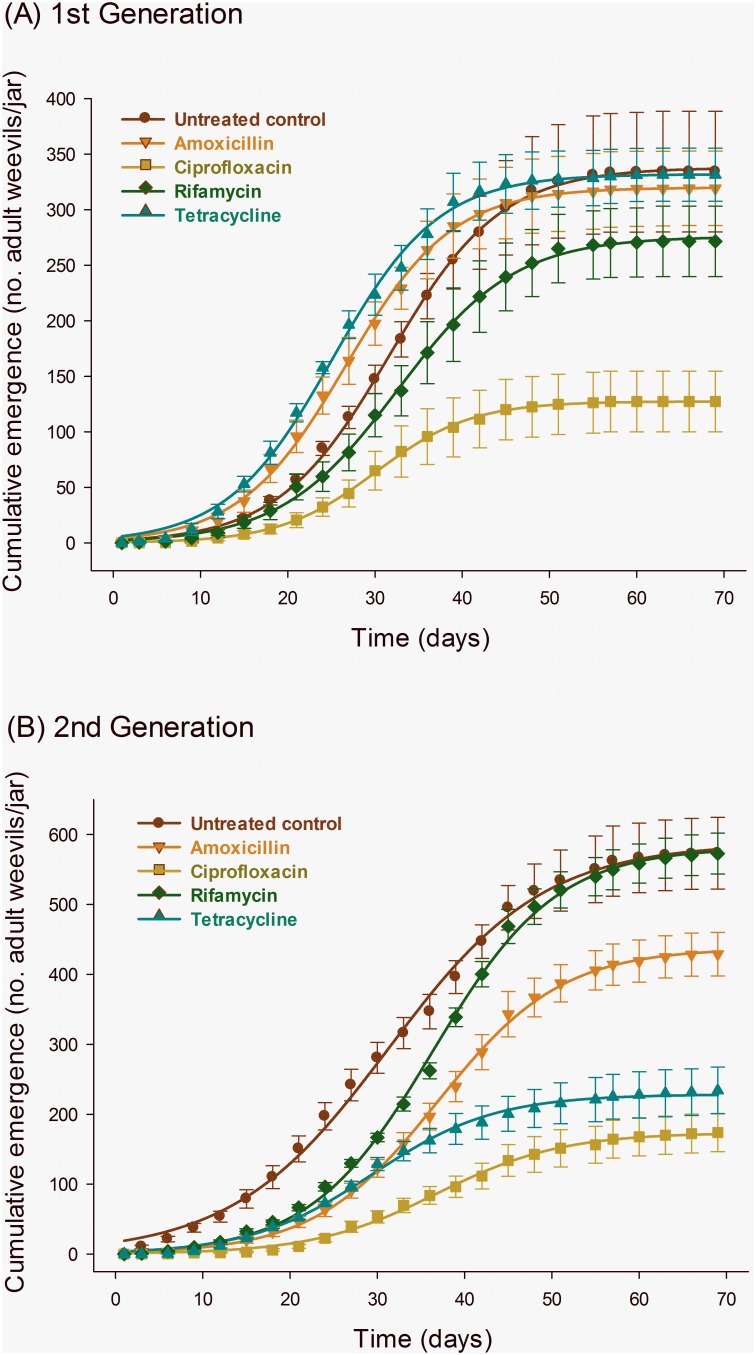
Cumulative emergence of F_1_ (A) and F_2_ progenies (B) of maize weevils (*Sitophilus zeamais*) exposed to different endosymbiont-reducing treatments. The symbols and vertical bars represent the means and standard errors of four replicates and the equation parameters are exhibited in Table 4.

The cumulative emergence profiles of endosymbiont-suppressed weevils are a direct consequence of the daily emergence. Ciprofloxacin again exhibited a consistent, substantial reduction in emergence for both F_1_ and F_2_ progenies, and intermediate results were observed with rifamycin for the F_1_ progeny. A trend reversal took place for amoxicillin and tetracycline, which exhibited reduced emergence only for the F_2_ weevil progeny (Table 4, Fig. 6).

**Table 4:** Summary of the non-linear regression analyses of the cumulative emergence curves (Fig. 5) of the F_1_ and F_2_ progenies of adult maize weevils (*Sitophilus zeamais*) exposed to different endosymbiont-suppression treatments via water-ingested antibiotics.

Generation	Model	Treatment	Parameter estimates (± SE)			df_error_	F	*P*	R^2^
			*a*	*b*	*c*				
		Untreated control	337.92±12.75	31.65±1.18	6.40±0.99	93	255.96	<0.001	0.85
	Sigmoid (3-parameter)	Amoxicillin	319. 91±8.08	26.67±0.85	6.07±0.72	93	407.98	<0.001	0.89
1^st^	y = *a/*(1+exp(−(x–*b*)/*c*))	Ciprofloxacin	127.35±6.81	30.06±1.65	5.51±1.40	93	105.11	<0.001	0.69
		Rifamycin	275±9.44	32.61±1.07	6.70±0.89	93	339.27	<0.001	0.88
		Tetracycline	331.98±6.05	25.14±0.63	6.05±0.53	93	715.02	<0.001	0.94
		Untreated control	587.37±15.65	31.25±0.93	8.92±0.74	93	679.42	<0.001	0.93
	Sigmoid (3-parameter)	Amoxicillin	437.49±10.19	36.96±0.68	7.12±0.55	93	1,046.67	<0.001	0.96
2^nd^	y = *a/*(1+exp(−(x–*b*)/*c*))	Ciprofloxacin	173.51±8.64	36.85±1.46	7.15±1.17	93	234.68	<0.001	0.83
		Rifamycin	581.50±8.78	36.39±0.44	7.06±0.36	93	2,359.23	<0.001	0.98
		Tetracycline	228.41±7.93	29.41±1.16	7.00±0.97	93	283.58	<0.001	0.86

All parameter estimates were significant at *P*<0.01 by Student’s *t*-test.

### Endosymbiont Load and Consequences

**Figure 7:**
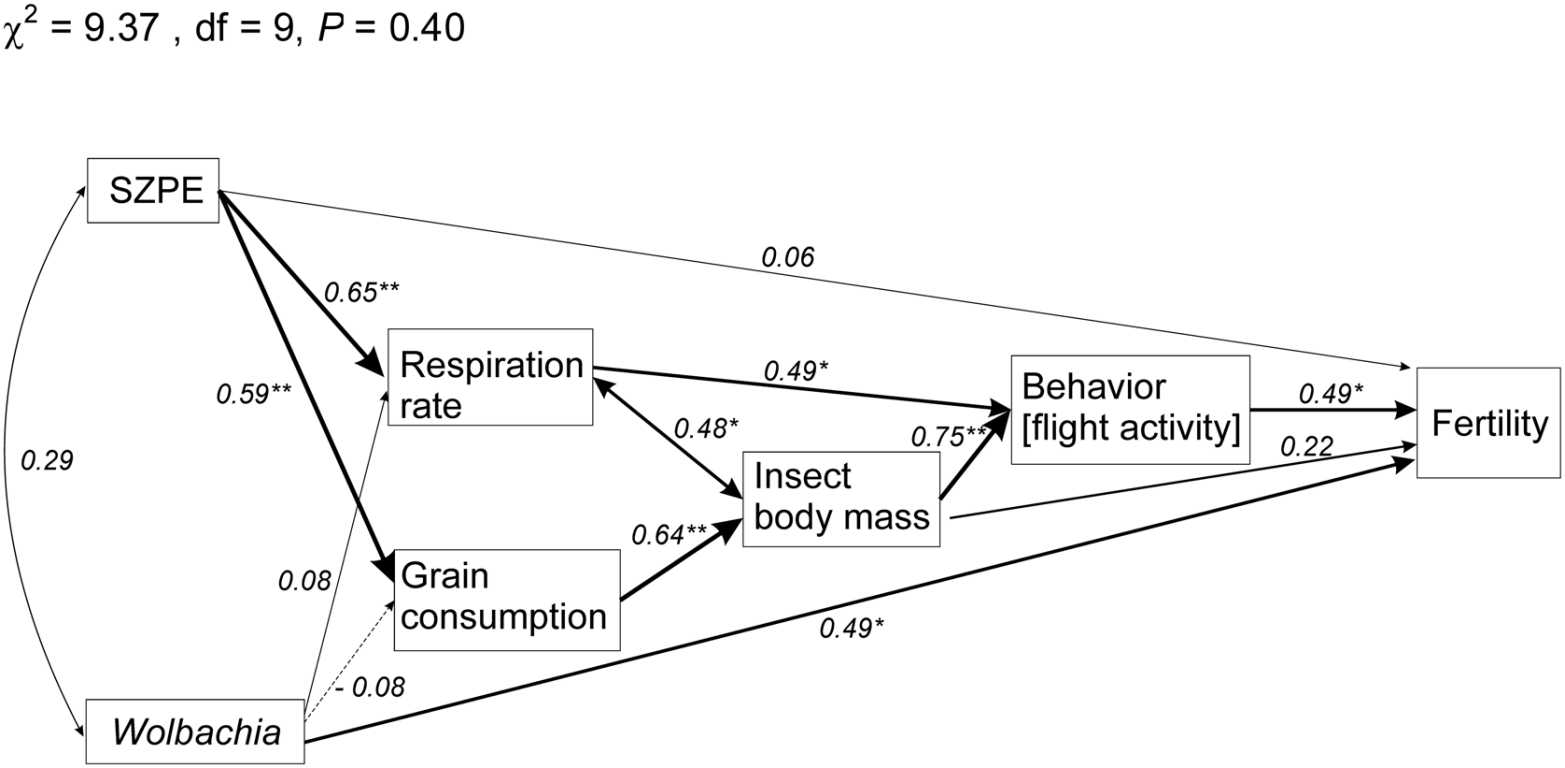
Path analysis diagram for the influence of the endosymbiont load of SZPE and *Wolbachia* on the respiration rate, grain consumption, body mass, behavior (flight activity, and fertility of the maize weevil (*Sitophilus zeamais*). The result of χ^2^ goodness-of-fit for the path model is indicated. One-headed arrows indicate causal relationships (regression), while doubled-headed arrows indicate correlation between the variables. Significance levels are represented by asterisks (*P<0.05, **P<0.01), and the thickness of each line is proportional to the strength of the relationship. Solid arrows indicate positive relationships, while dashed arrows indicate negative relationships. Direct, indirect and total values for path coefficients are fully presented in Table 5.

The consequences of the reduction of endosymbiont load in adult weevils (by providing antibiotics to the parental insects) were tracked using a hierarchical approach structured as a path diagram subjected to path analysis (Fig. 7). We expected that the endosymbiont load (SZPE and *Wolbachia*) would potentially influence respiration rate and body mass in addition to potential direct effects on reproduction, and lead to potential indirect effects on weevil behavior and reproduction. No significant departures from expected covariance matrices were observed in the hypothesized path diagram (χ^2^ = 9.37, df = 9, *P* = 0.40), indicating that the path model used is valid (Fig. 7).

The loads of SZPE and *Wolbachia* are not correlated, and only SZPE exhibited significant direct effects on both weevil respiration rate and grain consumption (Fig. 7, Table 5). The contribution of the *Wolbachia* load in both traits was negligible, although it exhibited a significant direct effect in insect fertility (Fig. 7, Table 5). Grain consumption exhibited a significant direct effect in weevil body mass (Fig. 7, Table 5). Weevil behavior, represented by the main behavioral trait (i.e., number of insects taking off for flight), differed among endosymbiont-reducing treatments and was significantly affected by body mass and respiration rate with indirect contributions by endosymbiont load and grain consumption (Table 5, Fig. 7). Among the traits assessed for the F_1_ progeny of endosymbiont-suppressed weevils subjected to antibiotic ingestion, only *Wolbachia* load and behavior had significant direct effects on the weevil reproductive output, but the whole of direct and indirect hypothesized effects in reproduction suggested in the path diagram were not significant (Fig. 7, Table 5).

**Table 5:** Direct (DE), indirect (IE), and total (TE) effects in the path diagram of Fig. 6 for the model on the influence of endosymbiont load and co-occurrence on the respiration rate, grain consumption, body mass, behavior (flight activity), and reproduction of the maize weevil *Sitophilus zeamais*.

Variable	Respirationrate (nmolCO_2_/insect/h)			Grainconsumption (g)			Insect bodymass (mg)			Behavior[flight activity](no. taking-offfor flight)			Fertility(total no.produced)		
	DE	IE	TE	DE	IE	TE	DE	IE	TE	DE	IE	TE	DE	IE	TE
SZPE(copies/µLDNA)	5.42×10^−4^	-	5.42×10^−4^	3.00×10^−4^	-	3.00×10^−4^	-	8.71×10^6^	8.71×10^6^	-	3.82×10^−5^	3.82×10^−5^	−0.002	0.001	−0.001
*Wolbachia*(copies/µLDNA)	−2.60×10^−3^	-	−2.60×10^−3^	−0.01	-	−0.01	-	−2.60×10^−4^	−2.60×10^−4^	-	−9.30×10^−4^	−9.30×10^−4^	0.26	−0.03	0.23
Respirationrate (nmolCO_2_/insect/h)	-	-	-	-	-	-	-	-	-	0.02	-	0.02	-	0.42	0.42
Grainconsumption (g)	-	-	-	-	-	-	0.03	-	0.03	-	0.09	0.09	-	3.19	3.19
Insect bodymass (mg)	-	-	-	-	-	-	-	-	-	3.10	-	3.10	49.05	63.37	112.42
Behavior[flightactivity](no. taking-off for flight)	-	-	-	-	-	-	-	-	-	-	-	-	20.42	-	20.42
R^2^	0.43			0.41			0.40			0.59			0.31		
*P*	0.002*			0.01*			0.002*			<0.001*			0.20		

Asterisks indicate significant differences at *P*<0.05.

## Discussion

Individual traits vary within a population, and the co-existence of varying loads of different endosymbiont species within an individual host makes understanding the impact of such associations in insect species even more difficult. The SPE association with weevils was recognized as early as the 1930s, while the facultative association between *Wolbachia* and weevils dates from the late 1990s [17]–[20]. The more intricate effects of SPE on weevil physiology, such as improved methionine metabolism, vitamin provision, energy metabolism and flight take-off were soon recognized upon full inactivation/suppression of the endosymbiont (i.e., using aposymbiotic weevils) [17], [28]–[30]. The recognition of the role of *Wolbachia* associated with grain weevils has been circumscribed to cytoplasmic incompatibility [18], [19], again using aposymbiotic weevils. Here we hypothesized that endosymbiont load and co-occurrence may interfere with weevil respiration rate, grain consumption, body mass, behavior, and reproduction.

Thermal treatment is the strategy usually employed to obtain aposymbiotic weevils, but tetracycline is also frequently used to suppress *Wolbachia* populations [17]–[19], [28]–[30]. Indeed the thermal treatment is very effective at fully inactivating not only SZPE but also *Wolbachia* in maize weevils. However, the thermally treated weevils obtained in our studies were unable to reproduce and were used only for parental determinations of respiration rate, body mass, and behavior. In contrast, the provision of antibiotics to maize weevils via ingested water was also effective at providing different endosymbiont loads of both SZPE and *Wolbachia*, allowing more comprehensive assessments up to the F_2_ progeny of treated individuals and demographic estimates and assessment of grain consumption. Therefore, the antibiotic-treated progeny was used to test our hypothesized relationship between endosymbiont load and co-occurrence and behavioral and physiological traits potentially affecting reproductive output.

Ciprofloxacin was particularly effective in suppressing SZPE, while tetracycline was fairly effective in suppressing *Wolbachia*, and thermal treatment simultaneously completely inactivated both SZPE and *Wolbachia* from their maize weevil hosts. The full simultaneous inactivation of both SZPE and *Wolbachia* significantly affected insect behavior and respiration rate, resembling the effect of the antibiotic ciprofloxacin that affected mainly SZPE, suggesting the pivotal involvement of this endosymbiont on weevil respiration and behavior, particularly flight and overall insect activity. These findings support earlier evidence of the intricate and important role of SPE in energy metabolism and flight take-off in grain weevils [17], [18], [29], [30]. The remaining antibiotics provided varying levels of suppression of both endosymbionts, allowing the correlations and regressions combined in our path diagram of effects.

*Wolbachia* load in the maize weevil was only a negligible direct contributor affecting respiration rate and grain consumption and indirectly affecting weevil body mass and behavior. However, *Wolbachia* load significantly affected weevil reproduction. Cytoplasmic incompatibility is frequently reported in arthropods [18], [19], [50], but our finding suggest that the effect of *Wolbachia* in weevils may go beyond that. *Wolbachia* also seems to potentiate the physiological and behavioral effects of SZPE in maize weevils, both directly (for respiration rate and grain consumption) and indirectly (for body mass and behavior), based on the direct and indirect effects evidenced in our path diagram. Furthermore, the complete suppression of *Wolbachia* and SZPE prevented maize weevil reproduction, although unfertilized eggs were laid by the thermally treated female weevils, suggesting a potentiation effect of the latter, with the former favoring reproductive output. Nonetheless, the thermal stress imposed on the insect may also have contributed to preventing their reproduction, considering that the progeny production was assessed in the thermally treated insects, unlike in the antibiotic-treated weevils, where the progeny was the target of the assessments.

SZPE load was of primary importance for the maize weevil, favoring higher respiration rate and grain consumption, which corresponded to improved gain in body mass in weevils with higher loads of this symbiont. The high body mass also exhibited a significant effect on insect behavior, particularly flight activity, aided by respiration rate. Earlier studies on the physiological role of SPE presence indicated involvement in nutrient provision and energy metabolism [17], [18], [29], [30]. Our results support this role and further indicate that such physiological effects are translated into gain in body mass and higher activity, particularly flight activity.

Although our path analysis did not provide evidence for increased overall progeny production in weevils with endosymbiont loads, the *Wolbachia* load positively affected fertility. Furthermore, daily progeny production was delayed with the reduction in endosymbiont load, particularly the drastic suppression of the SZPE load obtained with ciprofloxacin. This delayed progeny production had a negative effect on the weevil population growth, indicating an important reproductive role of SZPE in the maize weevil. Further evidence of *Wolbachia* and SZPE suppression leading to reproductive impairment is also provided by the inability of thermally treated maize weevils to reproduce (i.e., weevils with full inactivation of both SZPE and *Wolbachia*).

Our results with varying endosymbiont loads and co-occurrence of SZPE and *Wolbachia* in the maize weevil reinforce the notion of the relative independence of the symbionts, which are able to coexist, although the primary effects of the SZPE load in the host seem amplified by the *Wolbachia* load. The γ-Proteobacteria SPE, of which SZPE is a representative, is located in specific and differentiated cells (bacteriocytes) in bacteria-bearing tissue (bacteriome) found only in female germ cells and larval and ovarian bacteriomes [17]–[19]. This characteristic distribution of SPE in weevils likely maintains these endosymbionts in relative isolation, minimizing potential interactions with co-occurring symbionts such as *Wolbachia*. In contrast, *Wolbachia,* which is a α-Proteobacteria with facultative association in grain weevils, is disseminated throughout the body cells and at noticeably high densities in male and female germ cells, where it induces reproductive abnormalities [18], [19], [21], [22].

The co-occurrence of SZPE and *Wolbachia* in a key pest species of stored cereal grains, such as the maize weevil, has potential practical importance. An obvious possibility is the design of alternative management methods for the control of this pest species, such as sterile insect techniques (or incompatible insect techniques) and/or insertion of fitness reduction factors aiming at pest suppression or replacement [21], [22], [50]. These endosymbionts may prove important in strain divergence, with implications for grain loss and weevil control because endosymbiont load and co-occurrence affect grain consumption, consequently affecting grain loss and leading to higher economic losses. In addition, both endosymbiont load and co-occurrence affect insect activity, interfering with their dispersal and colonization, producing added potential consequences for pest control, which is variable between populations and even among individuals in a population [16], [44], [45]. Other unforeseeable consequences may also derive from variable endosymbiont loads and co-occurrence in arthropod pest species in general, and grain weevils in particular, which is likely to draw further attention in the future.

## Supporting Information

Figure S1 **Standard curve of** ***Wolbachia*** **16S gene in the presence of different concentrations (log) of the plasmid.**
(TIF)

Figure S2 **Standard curve of SZPE 16S gene in the presence of different concentrations (log) of the plasmid.**
(TIF)

Data S1 **Threshold cycle (Ct) values for** ***Wolbachia*** **16S gene from the F_1_ progenies of adult maize weevils (** ***Sitophilus zeamais*** **) exposed to different endosymbiont-suppression treatments.** Number of copies based on standard curve (y), number of copies corrected by the one-point calibration method (OPC) and number of copies per microliter of DNA.
(PDF)

Data S2 **Threshold cycle (Ct) values for** ***Wolbachia*** **16S gene from the F_2_ progenies of adult maize weevils (** ***Sitophilus zeamais*** **) exposed to different endosymbiont-suppression treatments.** Number of copies based on standard curve (y), number of copies corrected by the one-point calibration method (OPC) and number of copies per microliter of DNA.
(PDF)

Data S3 **Threshold cycle (Ct) values for gene SZPE 16S gene from the F_1_ progenies of adult maize weevils (** ***Sitophilus zeamais*** **) exposed to different endosymbiont-suppression treatments.** Number of copies based on standard curve (y), number of copies corrected by the one-point calibration method (OPC) and number of copies per microliter of DNA.
(PDF)

Data S4 **Threshold cycle (Ct) values for gene SZPE 16S gene from the F_2_ progenies of adult maize weevils (** ***Sitophilus zeamais*** **) exposed to different endosymbiont-suppression treatments.** Number of copies based on standard curve (y), number of copies corrected by the one-point calibration method (OPC) and number of copies per microliter of DNA.
(PDF)

Data S5 **Raw data of behavioral traits of F_1_ and F_2_ progenies of adult maize weevils (** ***Sitophilus zeamais*** **) exposed to different endosymbiont-suppression treatments.**
(PDF)

Data S6 **Raw data of respiration rate, body mass, grain consumption, and fertility of F_1_ and F_2_ progenies of adult maize weevils (** ***Sitophilus zeamais*** **) exposed to different endosymbiont-suppression treatments.**
(PDF)

Data S7 **Raw daily emergence data of 1^st^ generation insects.**
(PDF)

Data S8 **Raw daily emergence data of 2^nd^ generation insects.**
(PDF)

Data S9 **Raw cumulative emergence data of 1^st^ generations insects.**
(PDF)

Data S10 **Raw cumulative emergence data of 2^nd^ generation insects.**
(PDF)

Movie S1 **Video showing short recordings of each behavioral test.**
(MP4)
