# Supplementary figures and images for: Pleiotropic Impact of Endosymbiont Load and Co-Occurrence in the Maize Weevil Sitophilus zeamais
Source: PLoS One. 2014 Oct 27;9(10):e111396. doi: 10.1371/journal.pone.0111396 (PMC4210188; doi:10.1371/journal.pone.0111396)

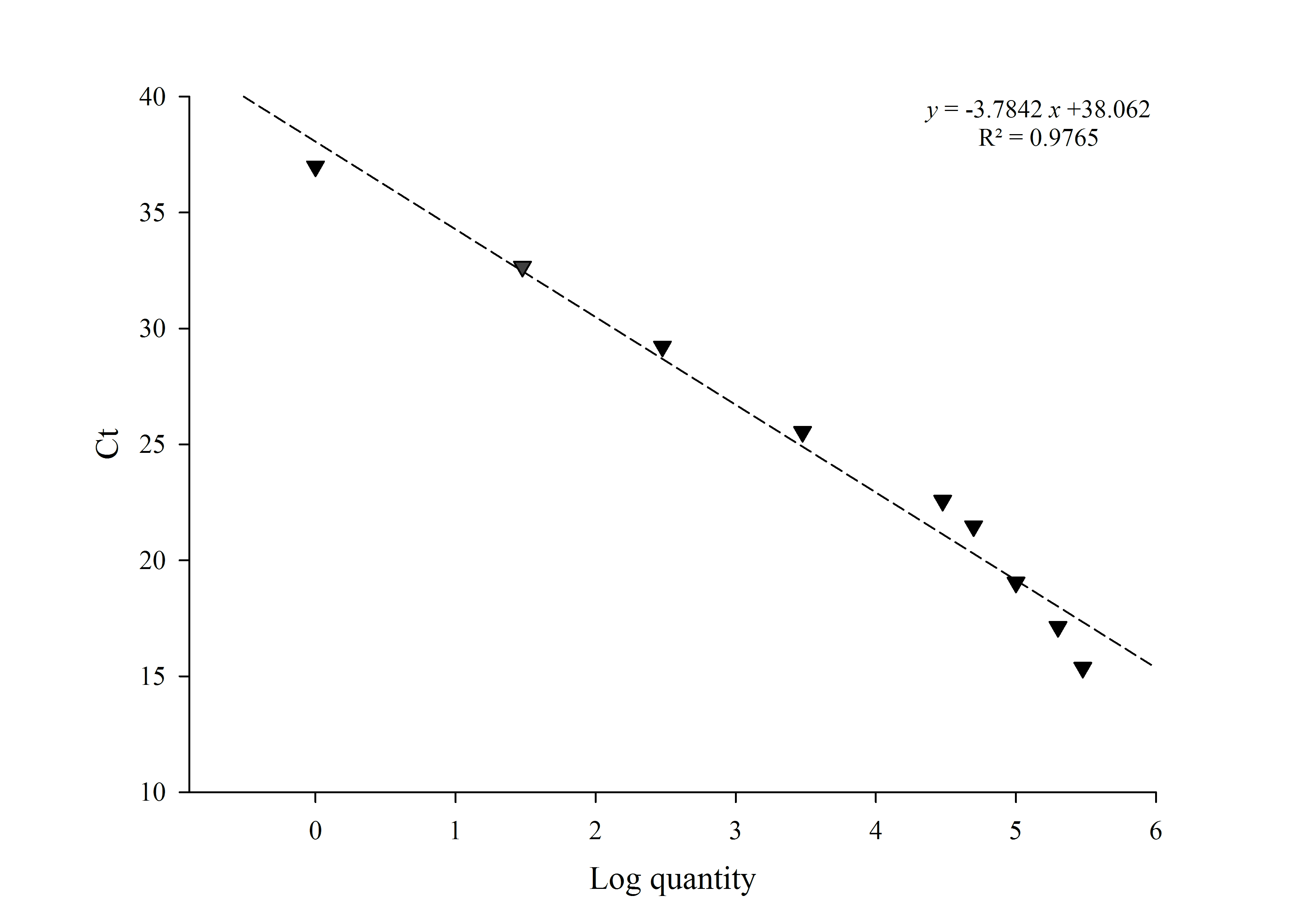

Supplement: Figure S1 — Standard curve of Wolbachia 16S gene in the presence of different concentrations (log) of the plasmid. (TIF) [file pone.0111396.s001.tif]

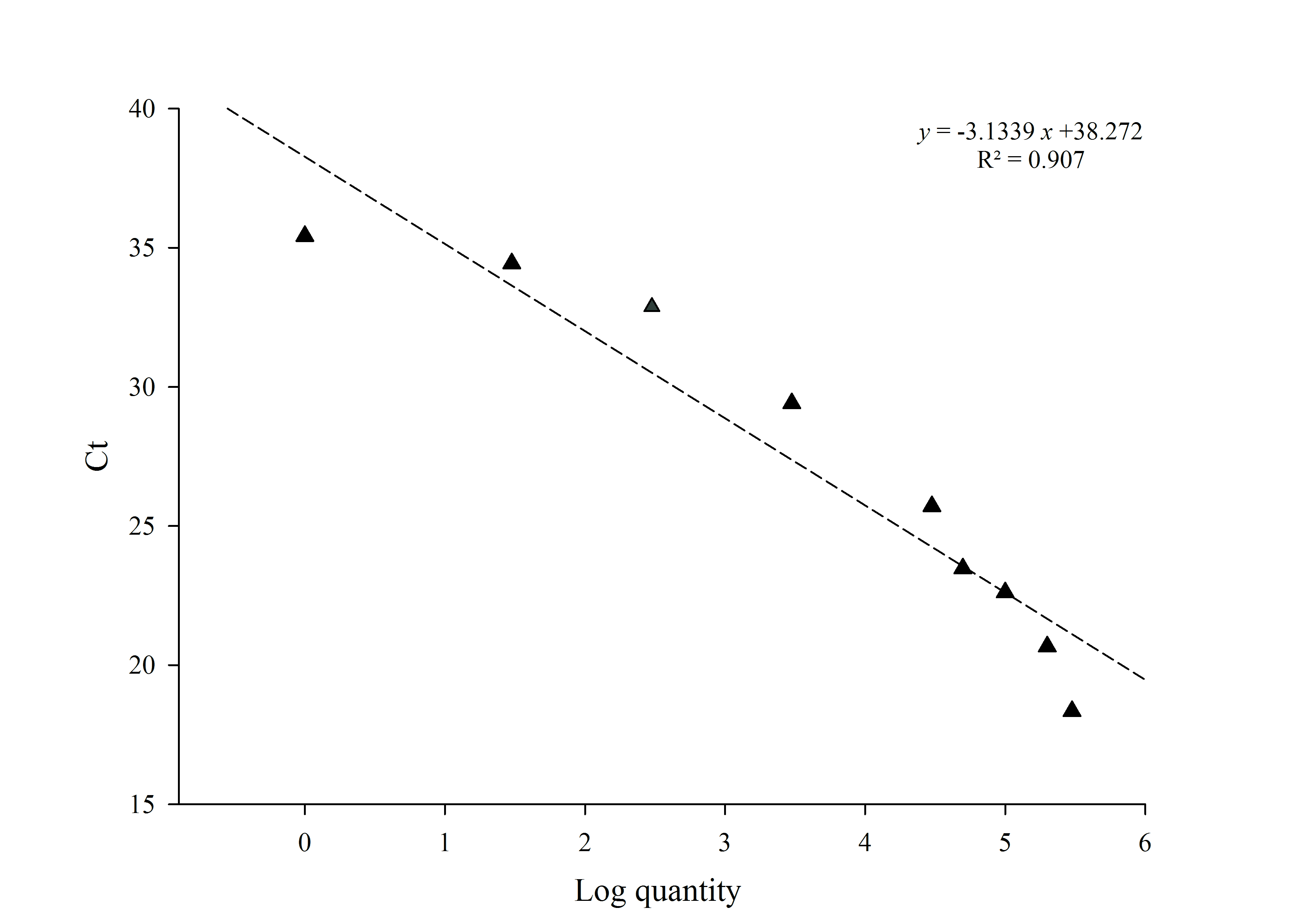

Supplement: Figure S2 — Standard curve of SZPE 16S gene in the presence of different concentrations (log) of the plasmid. (TIF) [file pone.0111396.s002.tif]
