# Supplementary material for: Pleiotropic Impact of Endosymbiont Load and Co-Occurrence in the Maize Weevil Sitophilus zeamais
Source: PLoS One. 2014 Oct 27;9(10):e111396. doi: 10.1371/journal.pone.0111396 (PMC4210188; doi:10.1371/journal.pone.0111396)
Supplement: Data S1 — Threshold cycle (Ct) values for Wolbachia 16S gene from the F1 progenies of adult maize weevils ( Sitophilus zeamais ) exposed to different endosymbiont-suppression treatments. Number of copies based on standard curve (y), number of copies corrected by the one-point calibration method (OPC) and number of copies per microliter of DNA. (PDF) [file pone.0111396.s003.pdf]

Sample	C_T	C_T	C_T	C_T Mean	C_T SD	*y*	OPC	copies/µL
Control	18.68	18.76	19.07	18.84	0.21	3.28	1911.38	159.28
Control	18.02	18.06	17.35	17.81	0.40	3.71	5132.36	427.70
Control	16.71	17.41	17.67	17.26	0.49	3.87	7452.74	621.06
Control	21.05	20.76	20.23	20.68	0.42	3.28	1902.92	158.58
Control	21.68	21.94	21.54	21.72	0.20	3.97	9308.28	775.69
Control	21.55	22.29	21.71	21.85	0.39	3.87	7461.30	621.77
Amoxicillin	26.92	26.47	27.09	26.82	0.32	3.03	1059.86	88.32
Amoxicillin	24.64	25.69	24.67	25.00	0.60	3.51	3220.53	268.38
Amoxicillin	25.19	25.85	25.70	25.58	0.34	3.35	2257.23	188.10
Amoxicillin	28.81	28.39	28.07	28.42	0.37	2.60	400.16	33.35
Amoxicillin	32.03	32.29	32.89	32.40	0.44	1.55	35.54	2.96
Amoxicillin	28.18	27.36	28.97	28.17	0.80	2.67	467.15	38.93
Ciprofloxacin	28.85	29.45	28.79	29.03	0.37	2.44	276.68	23.06
Ciprofloxacin	28.11	26.64	26.47	27.08	0.90	2.96	909.62	75.80
Ciprofloxacin	24.02	24.54	23.41	23.99	0.57	3.77	5944.93	495.41
Ciprofloxacin	29.86	29.27	31.13	30.09	0.95	2.16	145.65	12.14
Ciprofloxacin	27.05	26.01	25.65	26.24	0.73	3.18	1516.50	126.38
Ciprofloxacin	23.04	23.41	23.14	23.20	0.19	3.72	5243.53	436.96
Rifamycin	29.18	28.29	29.32	28.93	0.56	2.47	294.14	24.51
Rifamycin	29.35	31.69	31.37	30.81	1.27	1.97	93.96	7.83
Rifamycin	32.16	32.85	31.55	32.18	0.65	1.61	40.61	3.38
Rifamycin	29.01	29.26	29.32	29.20	0.16	2.40	250.26	20.86
Rifamycin	28.74	28.14	28.60	28.49	0.32	2.58	383.91	31.99
Rifamycin	33.44	34.02	33.96	33.81	0.32	1.18	15.12	1.26
Tetracycline	32.24	33.09	33.62	32.98	0.70	1.40	25.00	2.08
Tetracycline	33.24	34.22	34.03	33.83	0.52	1.17	14.93	1.24
Tetracycline	31.53	30.37	30.67	30.86	0.61	1.96	91.11	7.59
Tetracycline	28.03	27.68	28.11	27.94	0.23	2.73	537.47	44.79
Tetracycline	33.51	33.81	33.04	33.45	0.39	1.27	18.78	1.57
Tetracycline	33.98	33.09	33.71	33.60	0.45	1.24	17.21	1.43

Thermal treatment	33.98	33.61	33.15	33.58	0.41	1.18	15.27	1.27
Thermal treatment	32.09	32.03	32.44	32.18	0.22	1.55	35.73	2.98
Thermal treatment	33.71	33.76	33.76	33.74	0.03	1.14	13.84	1.15
Thermal treatment	35.75	35.75	35.97	35.82	0.12	0.59	3.90	0.33
Thermal treatment	35.91	35.95	35.13	35.67	0.46	0.63	4.30	0.36
Thermal treatment	35.60	35.95	35.56	35.71	0.21	0.62	4.20	0.35
