# Supplementary material for: Pleiotropic Impact of Endosymbiont Load and Co-Occurrence in the Maize Weevil Sitophilus zeamais
Source: PLoS One. 2014 Oct 27;9(10):e111396. doi: 10.1371/journal.pone.0111396 (PMC4210188; doi:10.1371/journal.pone.0111396)
Supplement: Data S2 — Threshold cycle (Ct) values for Wolbachia 16S gene from the F2 progenies of adult maize weevils ( Sitophilus zeamais ) exposed to different endosymbiont-suppression treatments. Number of copies based on standard curve (y), number of copies corrected by the one-point calibration method (OPC) and number of copies per microliter of DNA. (PDF) [file pone.0111396.s004.pdf]

Sample	C_T	C_T	C_T	C_T Mean	C_T SD	*y*	OPC	copies/µL
Control	26.46	26.07	26.30	26.28	0.20	3.11	1911.08	159.26
Control	25.47	25.00	25.05	25.17	0.26	3.41	512.39	42.70
Control	27.37	27.29	26.59	27.08	0.43	2.90	2412.83	201.07
Control	27.67	27.21	27.64	27.51	0.26	2.79	1018.45	84.87
Control	24.72	24.74	23.92	24.46	0.47	3.59	1823.53	151.96
Control	27.05	27.34	27.04	27.14	0.17	2.88	917.10	76.43
Amoxicillin	29.86	29.74	28.84	29.48	0.56	2.32	185.25	15.44
Amoxicillin	30.12	30.22	30.03	30.12	0.09	2.15	125.35	10.45
Amoxicillin	31.39	30.61	31.00	31.00	0.39	1.92	73.47	6.12
Amoxicillin	33.50	33.95	33.70	33.72	0.23	1.20	14.07	1.17
Amoxicillin	30.79	29.52	29.32	29.88	0.80	2.22	145.21	12.10
Amoxicillin	28.96	29.11	28.92	29.00	0.10	2.45	248.78	20.73
Ciprofloxacin	25.02	25.24	25.22	25.16	0.12	3.46	2566.44	213.87
Ciprofloxacin	28.89	29.44	28.77	29.03	0.36	2.44	242.98	20.25
Ciprofloxacin	30.40	30.10	29.33	29.94	0.55	2.20	139.78	11.65
Ciprofloxacin	25.97	26.71	25.88	26.19	0.46	3.19	1373.09	114.42
Ciprofloxacin	26.26	25.37	26.76	26.13	0.70	3.21	1423.51	118.63
Ciprofloxacin	26.68	27.27	26.01	26.65	0.63	3.07	1035.75	86.31
Rifamycin	28.36	28.08	28.89	28.44	0.41	2.60	348.15	29.01
Rifamycin	28.43	27.17	28.42	28.01	0.73	2.71	453.84	37.82
Rifamycin	31.37	31.03	30.98	31.13	0.21	1.89	67.98	5.67
Rifamycin	26.87	26.39	26.45	26.57	0.26	3.09	1086.94	90.58
Rifamycin	29.64	28.99	28.51	29.05	0.56	2.44	241.22	20.10
Rifamycin	26.60	25.89	25.85	26.11	0.42	3.21	1438.91	119.91
Tetracycline	31.21	31.77	30.41	31.13	0.68	1.89	67.84	5.65
Tetracycline	27.37	27.29	26.59	27.08	0.43	2.96	796.64	66.39
Tetracycline	38.48	37.06	38.26	37.94	0.76	0.09	1.08	0.09
Tetracycline	38.42	38.35	39.27	38.68	0.51	-0.11	0.69	0.06

Tetracycline	35.89	35.08	34.73	35.24	0.60	0.80	5.58	0.46
Tetracycline	35.98	34.61	35.29	35.29	0.69	0.79	5.39	0.45
