# Supplementary material for: Pleiotropic Impact of Endosymbiont Load and Co-Occurrence in the Maize Weevil Sitophilus zeamais
Source: PLoS One. 2014 Oct 27;9(10):e111396. doi: 10.1371/journal.pone.0111396 (PMC4210188; doi:10.1371/journal.pone.0111396)
Supplement: Data S3 — Threshold cycle (Ct) values for gene SZPE 16S gene from the F1 progenies of adult maize weevils ( Sitophilus zeamais ) exposed to different endosymbiont-suppression treatments. Number of copies based on standard curve (y), number of copies corrected by the one-point calibration method (OPC) and number of copies per microliter of DNA. (PDF) [file pone.0111396.s005.pdf]

**Sample**	**C_T**	**C_T**	**C_T**	**C_T Mean**	**C_T SD**	***y***	**OPC**	**copies/µL**
Control	19.87	19.96	19.14	19.66	0.45	5.52	331118.89	27593.24
Control	18.58	18.21	18.32	18.37	0.19	5.33	212273.99	17689.50
Control	19.43	18.99	18.76	19.06	0.34	5.74	555118.56	46259.88
Control	19.39	19.76	20.92	20.02	0.80	5.76	570759.61	47563.30
Control	19.87	18.37	19.68	19.31	0.82	5.35	222253.34	18521.11
Control	17.19	17.82	16.30	17.10	0.77	5.41	257617.92	21468.16
Amoxicillin	18.94	18.68	18.41	18.67	0.26	5.23	169499.38	14124.95
Amoxicillin	16.85	17.03	16.41	16.76	0.32	5.52	331118.89	27593.24
Amoxicillin	18.38	18.93	19.77	19.02	0.70	5.76	569871.92	47489.33
Amoxicillin	17.64	18.51	17.27	17.81	0.63	5.19	153570.43	12797.54
Amoxicillin	16.66	17.79	17.79	17.41	0.66	5.15	142039.78	11836.65
Amoxicillin	18.38	18.93	19.77	19.02	0.70	5.76	569871.92	47489.33
Ciprofloxacin	32.09	32.02	32.58	32.23	0.30	1.86	72.60	6.05
Ciprofloxacin	30.90	30.22	31.13	30.75	0.47	2.33	215.41	17.95
Ciprofloxacin	29.58	29.75	30.57	29.96	0.53	2.58	383.80	31.98
Ciprofloxacin	26.86	27.01	26.92	26.93	0.07	3.55	3567.37	297.28
Ciprofloxacin	35.87	35.87	35.11	35.62	0.44	0.78	6.02	0.50
Ciprofloxacin	35.13	35.06	34.80	35.00	0.18	0.98	9.51	0.79
Rifamycin	18.21	17.73	17.47	17.80	0.37	5.19	154161.10	12846.76
Rifamycin	17.44	16.19	17.23	16.95	0.67	5.24	172545.91	14378.83
Rifamycin	23.51	22.04	22.78	22.78	0.74	4.88	75357.22	6279.77
Rifamycin	15.09	15.73	14.94	15.25	0.42	5.63	422299.53	35191.63
Rifamycin	16.53	16.50	16.89	16.64	0.22	5.66	461653.94	38471.16
Rifamycin	22.94	22.38	22.24	22.52	0.37	4.96	90967.45	7580.62
Tetracycline	18.75	18.79	18.99	18.84	0.13	5.49	312320.37	26026.70
Tetracycline	17.07	17.35	17.67	17.36	0.30	5.39	246020.61	20501.72
Tetracycline	17.04	17.53	17.04	17.20	0.28	5.70	499301.13	41608.43
Tetracycline	17.23	17.54	17.71	17.49	0.24	5.29	193794.87	16149.57
Tetracycline	17.73	17.69	18.03	17.82	0.19	5.18	152627.90	12718.99
Tetracycline	16.10	16.03	15.84	15.99	0.13	5.61	403204.38	33600.37

Thermal treatment	34.86	35.63	35.24	35.24	0.39	1.09	1.98	0.91
Thermal treatment	35.37	35.37	35.30	35.33	0.02	1.11	1.17	0.92
Thermal treatment	31.74	29.97	30.86	30.86	1.39	2.30	199.16	16.60
Thermal treatment	37.28	37.39	37.34	37.34	1.97	0.23	1.71	0.14
Thermal treatment	34.58	34.90	34.74	34.74	1.00	1.06	11.49	0.96
Thermal treatment	33.13	32.95	33.04	33.04	0.94	1.60	40.08	3.34
