# Supplementary material for: Pleiotropic Impact of Endosymbiont Load and Co-Occurrence in the Maize Weevil Sitophilus zeamais
Source: PLoS One. 2014 Oct 27;9(10):e111396. doi: 10.1371/journal.pone.0111396 (PMC4210188; doi:10.1371/journal.pone.0111396)
Supplement: Data S4 — Threshold cycle (Ct) values for gene SZPE 16S gene from the F2 progenies of adult maize weevils ( Sitophilus zeamais ) exposed to different endosymbiont-suppression treatments. Number of copies based on standard curve (y), number of copies corrected by the one-point calibration method (OPC) and number of copies per microliter of DNA. (PDF) [file pone.0111396.s006.pdf]

**Sample**	**C_T**	**C_T**	**C_T**	**C_T Mean**	**C_T SD**	***y***	**OPC**	**copies/µL**
Control	19.05	19.18	19.17	19.13	0.07	5.00	100428.54	8369.05
Control	21.46	21.03	20.99	21.16	0.26	4.47	29300.27	2441.69
Control	20.98	19.45	18.92	19.78	1.07	4.83	67597.11	5633.09
Control	18.10	17.47	18.20	17.92	0.40	5.32	210045.60	17503.80
Control	20.56	21.99	20.78	21.11	0.77	4.48	30210.95	2517.58
Control	17.32	17.73	17.95	17.67	0.32	5.39	245459.91	20454.99
Amoxicillin	20.22	20.30	20.00	20.17	0.15	4.73	53428.97	4452.41
Amoxicillin	19.52	20.16	18.81	19.49	0.67	4.91	80655.38	6721.28
Amoxicillin	22.73	20.40	20.72	21.28	1.26	4.43	27170.53	2264.21
Amoxicillin	22.47	21.04	21.98	21.83	0.73	4.29	19465.96	1622.16
Amoxicillin	18.15	18.79	18.85	18.60	0.38	5.14	139254.75	11604.56
Amoxicillin	18.72	18.67	18.92	18.77	0.13	5.10	125210.69	10434.22
Ciprofloxacin	30.17	29.69	30.53	30.13	0.42	2.10	124.98	10.42
Ciprofloxacin	32.40	32.93	32.43	32.59	0.30	1.45	28.00	2.33
Ciprofloxacin	32.47	33.41	33.52	33.14	0.58	1.30	20.03	1.67
Ciprofloxacin	32.75	32.30	32.51	32.52	0.23	1.46	29.17	2.43
Ciprofloxacin	32.10	32.44	32.43	32.32	0.19	1.52	32.88	2.74
Ciprofloxacin	32.54	31.83	33.94	32.77	1.07	1.40	25.02	2.09
Rifamycin	22.15	22.56	22.17	22.30	0.23	4.17	14656.70	1221.39
Rifamycin	21.69	22.12	23.05	22.29	0.69	4.17	14760.41	1230.03
Rifamycin	23.96	23.19	23.12	23.42	0.46	3.87	7393.97	616.16
Rifamycin	21.67	21.55	21.54	21.59	0.07	4.35	22571.58	1880.96
Rifamycin	21.67	21.66	22.02	21.78	0.21	4.30	20024.50	1668.71
Rifamycin	26.77	28.70	27.97	27.81	0.97	2.71	511.33	42.61
Tetracycline	18.04	18.51	20.03	18.86	1.04	5.07	118708.03	9892.34
Tetracycline	20.71	22.99	20.61	21.44	1.35	4.39	24710.01	2059.17
Tetracycline	20.60	20.98	20.50	20.69	0.25	4.59	38912.84	3242.74
Tetracycline	19.44	18.58	18.46	18.83	0.53	5.08	120887.88	10073.99
Tetracycline	18.43	18.56	18.37	18.45	0.10	5.18	151890.78	12657.57

Tetracycline	18.13	18.30	18.44	18.29	0.16	5.23	168005.45	14000.45
