# Supplementary material for: Pleiotropic Impact of Endosymbiont Load and Co-Occurrence in the Maize Weevil Sitophilus zeamais
Source: PLoS One. 2014 Oct 27;9(10):e111396. doi: 10.1371/journal.pone.0111396 (PMC4210188; doi:10.1371/journal.pone.0111396)
Supplement: Data S5 — Raw data of behavioral traits of F1 and F2 progenies of adult maize weevils ( Sitophilus zeamais ) exposed to different endosymbiont-suppression treatments. (PDF) [file pone.0111396.s007.pdf]

Treatment	Distance walked (cm)	Resting time (s)	Walking velocity (cm/s)	Free-fall flight (nº)	Death-feigning (s)	Body righting (s)
Control	227.97	242.25	0.38	41.20	5.73	3.09
Control	243.02	239.07	0.41	44.70	5.04	3.70
Control	226.57	249.00	0.38	42.07	5.52	4.24
Control	286.51	178.12	0.48	33.67	6.16	2.50
Amoxicillin	209.13	264.67	0.35	6.60	6.04	4.41
Amoxicillin	250.57	207.59	0.42	7.50	5.98	4.34
Amoxicillin	228.41	244.95	0.38	7.00	7.30	5.11
Amoxicillin	202.81	270.47	0.34	6.90	6.34	3.79
Tetracycline	193.60	284.40	0.32	5.90	2.89	3.66
Tetracycline	212.09	255.84	0.35	7.00	3.37	3.37
Tetracycline	192.50	276.82	0.32	8.27	3.55	3.56
Tetracycline	198.97	260.87	0.33	7.50	3.49	3.05
Rifamycin	230.51	256.83	0.38	8.50	5.15	3.08
Rifamycin	184.41	291.33	0.31	8.30	4.19	2.98
Rifamycin	219.18	264.12	0.37	9.07	5.32	4.49
Rifamycin	204.41	262.25	0.34	12.27	6.99	3.75
Ciprofloxacin	198.71	288.92	0.33	5.20	20.07	6.75
Ciprofloxacin	185.05	280.07	0.31	5.40	16.01	8.58
Ciprofloxacin	195.56	269.96	0.33	4.20	15.30	7.33
Ciprofloxacin	229.49	221.30	0.38	6.60	12.31	7.59
Thermal treatment	183.93	285.66	0.31	5.00	13.01	7.03
Thermal treatment	191.92	268.15	0.32	6.30	5.82	5.56
Thermal treatment	197.38	269.97	0.33	5.80	6.47	6.04
Thermal treatment	181.49	297.25	0.30	5.90	6.15	7.98

Take-off flight (nº)	Height reached (cm)	Overall group activity (Δ pixels/s x 10^{-2})	Treatment
4.00	10.50	47.80	Control
3.00	13.33	52.91	Control
3.00	12.00	40.00	Control
3.00	17.33	42.70	Control
0.00	0.00	30.91	Amoxicillin
1.00	2.00	29.60	Amoxicillin
1.00	1.00	29.21	Amoxicillin
0.00	0.00	21.52	Amoxicillin
0.00	0.00	25.15	Tetracycline
0.00	0.00	14.44	Tetracycline
1.00	1.00	18.73	Tetracycline
0.00	0.00	31.61	Tetracycline
1.00	6.00	25.21	Rifamycin
2.00	8.00	23.10	Rifamycin
0.00	0.00	28.43	Rifamycin
2.00	3.50	24.73	Rifamycin
0.00	0.00	18.51	Ciprofloxacin
0.00	0.00	17.18	Ciprofloxacin
0.00	0.00	18.55	Ciprofloxacin
0.00	0.00	15.44	Ciprofloxacin
0.00	0.00	16.06	Thermal treatment
0.00	0.00	9.86	Thermal treatment
0.00	0.00	10.30	Thermal treatment
0.00	0.00	9.92	Thermal treatment
