# Supplementary material for: Pleiotropic Impact of Endosymbiont Load and Co-Occurrence in the Maize Weevil Sitophilus zeamais
Source: PLoS One. 2014 Oct 27;9(10):e111396. doi: 10.1371/journal.pone.0111396 (PMC4210188; doi:10.1371/journal.pone.0111396)
Supplement: Data S6 — Raw data of respiration rate, body mass, grain consumption, and fertility of F1 and F2 progenies of adult maize weevils ( Sitophilus zeamais ) exposed to different endosymbiont-suppression treatments. (PDF) [file pone.0111396.s008.pdf]

Treatment	Insect body mass 1st generation (mg)	Insect body mass 2nd generation (mg)	Respiration rate 1st generation (nmol CO_2/insect/h)	Respiration rate 2nd generation (nmol CO_2/insect/h)
Control	3.23	4.68	93.32	102.22
Control	3.22	4.11	93.31	89.71
Control	3.42	4.00	103.87	94.96
Control	3.52	3.87	101.14	99.76
Amoxicillin	2.76	2.81	80.99	83.52
Amoxicillin	2.68	3.18	72.63	76.92
Amoxicillin	2.72	3.20	77.30	76.04
Amoxicillin	2.93	3.38	82.56	78.66
Tetracycline	2.80	3.74	109.38	79.82
Tetracycline	2.87	2.70	85.18	82.98
Tetracycline	2.73	2.89	82.71	82.02
Tetracycline	2.98	3.20	84.63	83.42
Rifamycin	3.00	3.83	93.75	86.86
Rifamycin	2.54	3.26	77.25	84.82
Rifamycin	3.16	3.09	78.57	87.84
Rifamycin	2.96	3.56	83.59	83.61
Ciprofloxacin	2.60	2.46	75.95	73.65
Ciprofloxacin	2.40	2.89	77.35	75.39
Ciprofloxacin	2.38	2.45	70.09	56.43
Ciprofloxacin	2.50	2.39	71.99	64.07
Thermal treatment	3.03	—	85.12	—
Thermal treatment	3.23	—	75.06	—
Thermal treatment	2.79	—	76.85	—
Thermal treatment	3.04	—	88.22	—

Grain consumption 1st generation (g)	Grain consumption 2nd generation (g)	Fertility 1st generation (total no. produced)	Fertility 2nd generation (total no. produced)	Treatment
20.45	55.58	316.00	706.00	Control
19.10	51.42	264.00	570.00	Control
19.04	27.60	264.00	561.00	Control
23.70	28.88	493.00	456.00	Control
14.20	64.23	399.00	400.00	Amoxicillin
14.70	55.04	259.00	439.00	Amoxicillin
27.41	49.30	350.00	366.00	Amoxicillin
19.38	44.86	269.00	511.00	Amoxicillin
22.15	49.03	393.00	315.00	Tetracycline
19.41	24.95	329.00	164.00	Tetracycline
18.98	20.56	328.00	198.00	Tetracycline
14.45	28.04	276.00	259.00	Tetracycline
18.46	69.40	201.00	643.00	Rifamycin
20.93	45.84	235.00	590.00	Rifamycin
24.17	48.02	329.00	553.00	Rifamycin
19.19	55.89	321.00	505.00	Rifamycin
2.07	35.05	66.00	228.00	Ciprofloxacin
5.75	17.52	184.00	171.00	Ciprofloxacin
9.60	17.60	161.00	196.00	Ciprofloxacin
8.67	13.48	98.00	100.00	Ciprofloxacin
—	—	—	—	Thermal treatment
—	—	—	—	Thermal treatment
—	—	—	—	Thermal treatment
—	—	—	—	Thermal treatment
