# Supplementary material for: Pleiotropic Impact of Endosymbiont Load and Co-Occurrence in the Maize Weevil Sitophilus zeamais
Source: PLoS One. 2014 Oct 27;9(10):e111396. doi: 10.1371/journal.pone.0111396 (PMC4210188; doi:10.1371/journal.pone.0111396)
Supplement: Data S7 — Raw daily emergence data of 1st generation insects. (PDF) [file pone.0111396.s009.pdf]

1a Geração

Daily emergence

replicate	days	control	Amoxyllin	Cirpofluxacin	Rifamycin	Tetracycline
1	1	0	0	0	0	0
1	3	0	1	0	0	3
1	6	0	5	0	2	8
1	9	0	7	0	1	17
1	12	7	11	2	4	19
1	15	15	14	3	10	23
1	18	25	32	2	11	29
1	21	16	40	2	21	27
1	24	21	41	7	3	43
1	27	31	35	7	12	56
1	30	35	38	9	21	44
1	33	44	39	4	12	29
1	36	46	59	8	12	39
1	39	31	31	7	10	38
1	42	14	19	10	29	11
1	45	10	10	1	15	6
1	48	11	9	1	15	1
1	51	5	4	3	16	0
1	55	5	2	0	5	0
1	57	0	2	0	2	0
1	60	0	0	0	0	0
1	63	0	0	0	0	0
1	66	0	0	0	0	0
1	69	0	0	0	0	0
2	1	0	0	0	0	0
2	3	0	0	0	0	1
2	6	0	0	1	0	0
2	9	0	0	4	4	4
2	12	6	0	6	10	14
2	15	14	14	5	16	17
2	18	15	30	6	11	15
2	21	9	22	11	15	42
2	24	26	32	13	5	55
2	27	16	17	21	13	39
2	30	30	41	30	33	30
2	33	29	47	27	15	25
2	36	31	21	20	31	36
2	39	28	8	16	19	30
2	42	21	7	6	21	7
2	45	14	9	12	27	7
2	48	10	5	3	8	4
2	51	10	2	0	5	1
2	55	3	1	2	1	1
2	57	2	1	1	0	1
2	60	0	1	0	1	0
2	63	0	0	0	0	0

2	**66**	0	1	0	0	0
2	**69**	0	0	0	0	0
3	**1**	0	0	0	0	0
3	**3**	0	0	0	0	1
3	**6**	0	7	0	0	2
3	**9**	0	13	1	3	7
3	**12**	2	15	4	0	17
3	**15**	14	28	4	2	23
3	**18**	13	38	8	2	36
3	**21**	23	29	14	13	42
3	**24**	33	40	16	11	37
3	**27**	32	40	17	27	42
3	**30**	28	27	29	38	22
3	**33**	29	23	27	31	22
3	**36**	30	33	13	54	20
3	**39**	19	21	5	41	24
3	**42**	14	9	7	30	11
3	**45**	12	14	8	22	10
3	**48**	6	7	1	20	3
3	**51**	3	1	5	21	1
3	**55**	3	4	2	5	1
3	**57**	3	1	0	4	3
3	**60**	0	0	0	1	3
3	**63**	0	0	0	4	1
3	**66**	0	0	0	0	0
3	**69**	0	0	0	0	0
4	**1**	0	0	0	0	0
4	**3**	0	0	0	2	0
4	**6**	0	2	0	1	1
4	**9**	2	6	0	4	5
4	**12**	9	11	0	6	14
4	**15**	18	16	1	8	36
4	**18**	12	18	3	18	33
4	**21**	26	25	5	38	33
4	**24**	34	34	11	18	27
4	**27**	33	33	4	35	18
4	**30**	44	28	14	42	12
4	**33**	42	18	11	30	21
4	**36**	49	25	13	40	26
4	**39**	51	26	5	31	24
4	**42**	50	9	7	21	10
4	**45**	54	6	13	7	3
4	**48**	34	4	5	7	4
4	**51**	16	1	1	10	3
4	**55**	12	2	2	2	2
4	**57**	3	3	3	1	2
4	**60**	2	1	0	0	2
4	**63**	2	1	0	0	0
4	**66**	0	0	0	0	0
4	**69**	0	0	0	0	0
