# Supplementary material for: Pleiotropic Impact of Endosymbiont Load and Co-Occurrence in the Maize Weevil Sitophilus zeamais
Source: PLoS One. 2014 Oct 27;9(10):e111396. doi: 10.1371/journal.pone.0111396 (PMC4210188; doi:10.1371/journal.pone.0111396)
Supplement: Data S8 — Raw daily emergence data of 2nd generation insects. (PDF) [file pone.0111396.s010.pdf]

## 2a Geração

Daily emergence

| replicate | days | control | Amoxyllin | Cirpofluxacin | Rifamycin | Tetracycline |
|---|---|---|---|---|---|---|
| 1 | 1 | 3 | 0 | 0 | 0 | 0 |
| 1 | 3 | 7 | 3 | 0 | 1 | 0 |
| 1 | 6 | 6 | 2 | 0 | 0 | 1 |
| 1 | 9 | 10 | 4 | 0 | 3 | 1 |
| 1 | 12 | 11 | 8 | 0 | 5 | 6 |
| 1 | 15 | 12 | 5 | 1 | 12 | 13 |
| 1 | 18 | 23 | 9 | 5 | 18 | 17 |
| 1 | 21 | 38 | 9 | 6 | 19 | 12 |
| 1 | 24 | 41 | 12 | 14 | 23 | 18 |
| 1 | 27 | 42 | 28 | 12 | 36 | 21 |
| 1 | 30 | 57 | 21 | 19 | 40 | 38 |
| 1 | 33 | 53 | 25 | 21 | 49 | 30 |
| 1 | 36 | 55 | 32 | 20 | 63 | 30 |
| 1 | 39 | 81 | 35 | 16 | 74 | 28 |
| 1 | 42 | 63 | 47 | 22 | 63 | 14 |
| 1 | 45 | 69 | 42 | 39 | 93 | 18 |
| 1 | 48 | 42 | 42 | 17 | 37 | 17 |
| 1 | 51 | 31 | 31 | 13 | 33 | 16 |
| 1 | 55 | 29 | 23 | 6 | 32 | 13 |
| 1 | 57 | 19 | 10 | 7 | 15 | 7 |
| 1 | 60 | 4 | 3 | 3 | 8 | 7 |
| 1 | 63 | 5 | 7 | 3 | 9 | 4 |
| 1 | 66 | 2 | 2 | 4 | 6 | 1 |
| 1 | 69 | 3 | 0 | 0 | 4 | 3 |
| 2 | 1 | 4 | 0 | 0 | 0 | 0 |
| 2 | 3 | 5 | 1 | 0 | 0 | 0 |
| 2 | 6 | 11 | 1 | 1 | 5 | 0 |
| 2 | 9 | 17 | 2 | 1 | 9 | 2 |
| 2 | 12 | 16 | 2 | 1 | 8 | 5 |
| 2 | 15 | 20 | 4 | 0 | 13 | 15 |
| 2 | 18 | 25 | 7 | 2 | 13 | 14 |
| 2 | 21 | 31 | 13 | 6 | 29 | 20 |
| 2 | 24 | 49 | 16 | 9 | 32 | 19 |
| 2 | 27 | 38 | 25 | 16 | 30 | 16 |
| 2 | 30 | 28 | 36 | 12 | 43 | 17 |
| 2 | 33 | 26 | 47 | 16 | 60 | 6 |
| 2 | 36 | 21 | 40 | 10 | 48 | 10 |
| 2 | 39 | 57 | 41 | 11 | 85 | 6 |
| 2 | 42 | 76 | 57 | 15 | 71 | 6 |
| 2 | 45 | 70 | 70 | 26 | 71 | 8 |
| 2 | 48 | 21 | 15 | 8 | 23 | 5 |
| 2 | 51 | 16 | 18 | 6 | 17 | 3 |
| 2 | 55 | 16 | 16 | 8 | 7 | 1 |
| 2 | 57 | 11 | 11 | 13 | 8 | 5 |
| 2 | 60 | 4 | 3 | 5 | 6 | 1 |

| | | | | | | |
|---|---|---|---|---|---|---|
| 2 | **63** | 4 | 10 | 0 | 8 | 1 |
| 2 | **66** | 3 | 4 | 4 | 4 | 2 |
| 2 | **69** | 1 | 0 | 1 | 0 | 2 |
| 3 | **1** | 1 | 0 | 0 | 0 | 0 |
| 3 | **3** | 4 | 1 | 0 | 2 | 0 |
| 3 | **6** | 12 | 0 | 0 | 4 | 0 |
| 3 | **9** | 15 | 0 | 3 | 4 | 5 |
| 3 | **12** | 18 | 5 | 0 | 6 | 8 |
| 3 | **15** | 31 | 11 | 3 | 18 | 8 |
| 3 | **18** | 40 | 13 | 4 | 8 | 15 |
| 3 | **21** | 53 | 17 | 7 | 18 | 10 |
| 3 | **24** | 51 | 19 | 17 | 33 | 23 |
| 3 | **27** | 67 | 26 | 26 | 31 | 22 |
| 3 | **30** | 48 | 32 | 15 | 33 | 35 |
| 3 | **33** | 36 | 30 | 17 | 38 | 13 |
| 3 | **36** | 32 | 32 | 18 | 41 | 3 |
| 3 | **39** | 26 | 47 | 17 | 81 | 11 |
| 3 | **42** | 34 | 33 | 18 | 73 | 6 |
| 3 | **45** | 33 | 36 | 16 | 58 | 14 |
| 3 | **48** | 24 | 24 | 8 | 30 | 3 |
| 3 | **51** | 7 | 14 | 9 | 21 | 7 |
| 3 | **55** | 9 | 12 | 1 | 21 | 3 |
| 3 | **57** | 10 | 2 | 8 | 8 | 3 |
| 3 | **60** | 4 | 6 | 4 | 11 | 1 |
| 3 | **63** | 4 | 3 | 3 | 8 | 3 |
| 3 | **66** | 2 | 2 | 0 | 3 | 2 |
| 3 | **69** | 0 | 1 | 2 | 3 | 3 |
| 4 | **1** | 3 | 1 | 0 | 0 | 0 |
| 4 | **3** | 14 | 2 | 0 | 0 | 0 |
| 4 | **6** | 16 | 3 | 1 | 4 | 0 |
| 4 | **9** | 22 | 6 | 1 | 7 | 8 |
| 4 | **12** | 19 | 9 | 0 | 11 | 11 |
| 4 | **15** | 39 | 11 | 0 | 17 | 7 |
| 4 | **18** | 36 | 18 | 0 | 15 | 18 |
| 4 | **21** | 40 | 20 | 2 | 17 | 12 |
| 4 | **24** | 45 | 20 | 6 | 31 | 23 |
| 4 | **27** | 32 | 30 | 12 | 37 | 39 |
| 4 | **30** | 23 | 38 | 11 | 32 | 36 |
| 4 | **33** | 26 | 42 | 11 | 44 | 25 |
| 4 | **36** | 15 | 49 | 9 | 39 | 17 |
| 4 | **39** | 34 | 48 | 6 | 66 | 22 |
| 4 | **42** | 29 | 60 | 6 | 39 | 10 |
| 4 | **45** | 22 | 69 | 8 | 51 | 10 |
| 4 | **48** | 8 | 15 | 2 | 24 | 6 |
| 4 | **51** | 7 | 18 | 7 | 24 | 4 |
| 4 | **55** | 10 | 23 | 5 | 16 | 4 |
| 4 | **57** | 7 | 10 | 4 | 9 | 1 |
| 4 | **60** | 6 | 8 | 2 | 10 | 2 |
| 4 | **63** | 2 | 4 | 3 | 6 | 1 |
| 4 | **66** | 1 | 5 | 1 | 4 | 1 |

| | | | | | | |
|---|---|---|---|---|---|---|
| 4 | **69** | 0 | 2 | 3 | 2 | 2 |
