# Supplementary material for: Pleiotropic Impact of Endosymbiont Load and Co-Occurrence in the Maize Weevil Sitophilus zeamais
Source: PLoS One. 2014 Oct 27;9(10):e111396. doi: 10.1371/journal.pone.0111396 (PMC4210188; doi:10.1371/journal.pone.0111396)
Supplement: Data S9 — Raw cumulative emergence data of 1st generations insects. (PDF) [file pone.0111396.s011.pdf]

1a Geração

Cumulative emergence

replicate	days	control	Amoxyllin	Cirpofluxacin	Rifamycin	Tetracycline
1	1	0	0	0	0	0
1	3	0	1	0	0	3
1	6	0	6	0	2	11
1	9	0	13	0	3	28
1	12	7	24	2	7	47
1	15	22	38	5	17	70
1	18	47	70	7	28	99
1	21	63	110	9	49	126
1	24	84	151	16	52	169
1	27	115	186	23	64	225
1	30	150	224	32	85	269
1	33	194	263	36	97	298
1	36	240	322	44	109	337
1	39	271	353	51	119	375
1	42	285	372	61	148	386
1	45	295	382	62	163	392
1	48	306	391	63	178	393
1	51	311	395	66	194	393
1	55	316	397	66	199	393
1	57	316	399	66	201	393
1	60	316	399	66	201	393
1	63	316	399	66	201	393
1	66	316	399	66	201	393
1	69	316	399	66	201	393
2	1	0	0	0	0	0
2	3	0	0	0	0	1
2	6	0	0	1	0	1
2	9	0	0	5	4	5
2	12	6	0	11	14	19
2	15	20	14	16	30	36
2	18	35	44	22	41	51
2	21	44	66	33	56	93
2	24	70	98	46	61	148
2	27	86	115	67	74	187
2	30	116	156	97	107	217
2	33	145	203	124	122	242
2	36	176	224	144	153	278
2	39	204	232	160	172	308
2	42	225	239	166	193	315
2	45	239	248	178	220	322
2	48	249	253	181	228	326
2	51	259	255	181	233	327
2	55	262	256	183	234	328
2	57	264	257	184	234	329
2	60	264	258	184	235	329
2	63	264	258	184	235	329

2	**66**	264	259	184	235	329
2	**69**	264	259	184	235	329
3	**1**	0	0	0	0	0
3	**3**	0	0	0	0	1
3	**6**	0	7	0	0	3
3	**9**	0	20	1	3	10
3	**12**	2	35	5	3	27
3	**15**	16	63	9	5	50
3	**18**	29	101	17	7	86
3	**21**	52	130	31	20	128
3	**24**	85	170	47	31	165
3	**27**	117	210	64	58	207
3	**30**	145	237	93	96	229
3	**33**	174	260	120	127	251
3	**36**	204	293	133	181	271
3	**39**	223	314	138	222	295
3	**42**	237	323	145	252	306
3	**45**	249	337	153	274	316
3	**48**	255	344	154	294	319
3	**51**	258	345	159	315	320
3	**55**	261	349	161	320	321
3	**57**	264	350	161	324	324
3	**60**	264	350	161	325	327
3	**63**	264	350	161	329	328
3	**66**	264	350	161	329	328
3	**69**	264	350	161	329	328
4	**1**	0	0	0	0	0
4	**3**	0	0	0	2	0
4	**6**	0	2	0	3	1
4	**9**	2	8	0	7	6
4	**12**	11	19	0	13	20
4	**15**	29	35	1	21	56
4	**18**	41	53	4	39	89
4	**21**	67	78	9	77	122
4	**24**	101	112	20	95	149
4	**27**	134	145	24	130	167
4	**30**	178	173	38	172	179
4	**33**	220	191	49	202	200
4	**36**	269	216	62	242	226
4	**39**	320	242	67	273	250
4	**42**	370	251	74	294	260
4	**45**	424	257	87	301	263
4	**48**	458	261	92	308	267
4	**51**	474	262	93	318	270
4	**55**	486	264	95	320	272
4	**57**	489	267	98	321	274
4	**60**	491	268	98	321	276
4	**63**	493	269	98	321	276
4	**66**	493	269	98	321	276
4	**69**	493	269	98	321	276
