# Supplementary material for: Pleiotropic Impact of Endosymbiont Load and Co-Occurrence in the Maize Weevil Sitophilus zeamais
Source: PLoS One. 2014 Oct 27;9(10):e111396. doi: 10.1371/journal.pone.0111396 (PMC4210188; doi:10.1371/journal.pone.0111396)
Supplement: Data S10 — Raw cumulative emergence data of 2nd generation insects. (PDF) [file pone.0111396.s012.pdf]

2a Geração

Cumulative emergence

replicate	days	control	Amoxyllin	Cirpofluxacin	Rifamycin	Tetracycline
**1**	**1**	0	0	0	0	0
**1**	**3**	10	3	0	1	0
**1**	**6**	16	5	0	1	1
**1**	**9**	26	9	0	4	2
**1**	**12**	37	17	0	9	8
**1**	**15**	49	22	1	21	21
**1**	**18**	72	31	6	39	38
**1**	**21**	110	40	12	58	50
**1**	**24**	151	52	26	81	68
**1**	**27**	193	80	38	117	89
**1**	**30**	250	101	57	157	127
**1**	**33**	303	126	78	206	157
**1**	**36**	358	158	98	269	187
**1**	**39**	439	193	114	343	215
**1**	**42**	502	240	136	406	229
**1**	**45**	571	282	175	499	247
**1**	**48**	613	324	192	536	264
**1**	**51**	644	355	205	569	280
**1**	**55**	673	378	211	601	293
**1**	**57**	692	388	218	616	300
**1**	**60**	696	391	221	624	307
**1**	**63**	701	398	224	633	311
**1**	**66**	703	400	228	639	312
**1**	**69**	706	400	228	643	315
2	**1**	**0**	0	0	0	0
2	**3**	**9**	1	0	0	0
2	**6**	**20**	2	1	5	0
2	**9**	**37**	4	2	14	2
2	**12**	**53**	6	3	22	7
2	**15**	**73**	10	3	35	22
2	**18**	**98**	17	5	48	36
2	**21**	**129**	30	11	77	56
2	**24**	**178**	46	20	109	75
2	**27**	**216**	71	36	139	91
2	**30**	**244**	107	48	182	108
2	**33**	**270**	154	64	242	114
2	**36**	**291**	194	74	290	124
2	**39**	**348**	235	85	375	130
2	**42**	**424**	292	100	446	136
2	**45**	**494**	362	126	517	144
2	**48**	**515**	377	134	540	149
2	**51**	**531**	395	140	557	152
2	**55**	**547**	411	148	564	153
2	**57**	**558**	422	161	572	158
2	**60**	**562**	425	166	578	159

2	**63**	**566**	435	166	586	160
2	**66**	**569**	439	170	590	162
2	**69**	570	439	171	590	164
3	**1**	0	0	0	0	0
3	**3**	5	1	0	2	0
3	**6**	17	1	0	6	0
3	**9**	32	1	3	10	5
3	**12**	50	6	3	16	13
3	**15**	81	17	6	34	21
3	**18**	121	30	10	42	36
3	**21**	174	47	17	60	46
3	**24**	225	66	34	93	69
3	**27**	292	92	60	124	91
3	**30**	340	124	75	157	126
3	**33**	376	154	92	195	139
3	**36**	408	186	110	236	142
3	**39**	434	233	127	317	153
3	**42**	468	266	145	390	159
3	**45**	501	302	161	448	173
3	**48**	525	326	169	478	176
3	**51**	532	340	178	499	183
3	**55**	541	352	179	520	186
3	**57**	551	354	187	528	189
3	**60**	555	360	191	539	190
3	**63**	559	363	194	547	193
3	**66**	561	365	194	550	195
3	**69**	561	366	196	553	198
4	**1**	0	0	0	0	0
4	**3**	17	3	0	0	0
4	**6**	33	6	1	4	0
4	**9**	55	12	2	11	8
4	**12**	74	21	2	22	19
4	**15**	113	32	2	39	26
4	**18**	149	50	2	54	44
4	**21**	189	70	4	71	56
4	**24**	234	90	10	102	79
4	**27**	266	120	22	139	118
4	**30**	289	158	33	171	154
4	**33**	315	200	44	215	179
4	**36**	330	249	53	254	196
4	**39**	364	297	59	320	218
4	**42**	393	357	65	359	228
4	**45**	415	426	73	410	238
4	**48**	423	441	75	434	244
4	**51**	430	459	82	458	248
4	**55**	440	482	87	474	252
4	**57**	447	492	91	483	253
4	**60**	453	500	93	493	255
4	**63**	455	504	96	499	256
4	**66**	456	509	97	503	257

4	**69**	456	511	100	505	259
